# Human MASLD is a diurnal disease driven by multisystem insulin resistance and reduced insulin availability at night

**DOI:** 10.1016/j.cmet.2025.12.004

**Published:** 2026-01-12

**Authors:** Thomas Marjot, Kieran Smith, Felix Westcott, Sarah White, Elspeth Johnson, Nikola Srnic, Amy Barrett, Ellis Hall, Kate Gralton, Kaitlyn Dennis, Hamish Miller, Riccardo Pofi, Jeremy F.L. Cobbold, Rebecca Richmond, Fredrik Karpe, Ronnie Blazev, Matthew J. Watt, Benjamin L. Parker, Leanne Hodson, David W. Ray, Jeremy W. Tomlinson

**Affiliations:** 1 Oxford Centre for Diabetes Endocrinology and Metabolism (OCDEM), Churchill Hospital, Radcliffe Department of Medicine, University of Oxford, Oxford, UK; 2 Translational Gastroenterology and Liver Unit (TGLU), Nuffield Department of Medicine, John Radcliffe Hospital, University of Oxford, Oxford, UK; 3 NIHR Oxford Biomedical Research Centre, University of Oxford, Oxford, UK; 4 NIHR Oxford Health Biomedical Research Centre, University of Oxford, Oxford, UK; 5 MRC Integrative Epidemiology Unit, Bristol Medical School, University of Bristol, Bristol, UK; 6 Department of Anatomy & Physiology, School of Biomedical Sciences, Faculty of Medicine Dentistry and Health Sciences, University of Melbourne, Parkville, VIC, Australia; 7 Sir Jules Thorn Sleep and Circadian Neuroscience Institute (SCNi), University of Oxford, Oxford, UK

## Abstract

Hepatic lipid and glucose metabolism have been shown to be under tight circadian control in pre-clinical models. However, it remains unknown whether diurnal patterns exist in functional processes governing intra-hepatic lipid accumulation in humans. We performed metabolic phenotyping, including state-of-the-art stable isotope techniques, during day and night in patients with metabolic dysfunction-associated steatotic liver disease (MASLD) and overweight controls (NCT05962099). The primary outcome was diurnal change in hepatic *de novo* lipogenesis (DNL), alongside a number of secondary outcomes, including changes in hepatic glucose production, glucose disposal, plasma non-esterified fatty acids (NEFAs), and whole-body glucose and lipid oxidation. We show that nighttime metabolic dysfunction is a hallmark of MASLD with multiple pathogenic pathways upregulated at night, including hepatic and peripheral insulin resistance, DNL, and systemic NEFA exposure. Insulin resistance is compounded by lower plasma insulin levels at night, secondary to reduced insulin secretion and elevated insulin clearance. Diurnal differences persist when performing identical investigations after weight loss with liver fat reductions, suggesting that nighttime metabolic dysfunction may be a primary driver of steatosis. These findings will help establish the optimal window for energy intake, exercise, and medication delivery in patients with MASLD. Integrated proteomics of plasma, adipose, and skeletal muscle tissue across day and night also identified a number of specific molecular targets that may offer therapeutic potential in the treatment of metabolic disease.

## Graphical abstract

**Graphical abstract:**
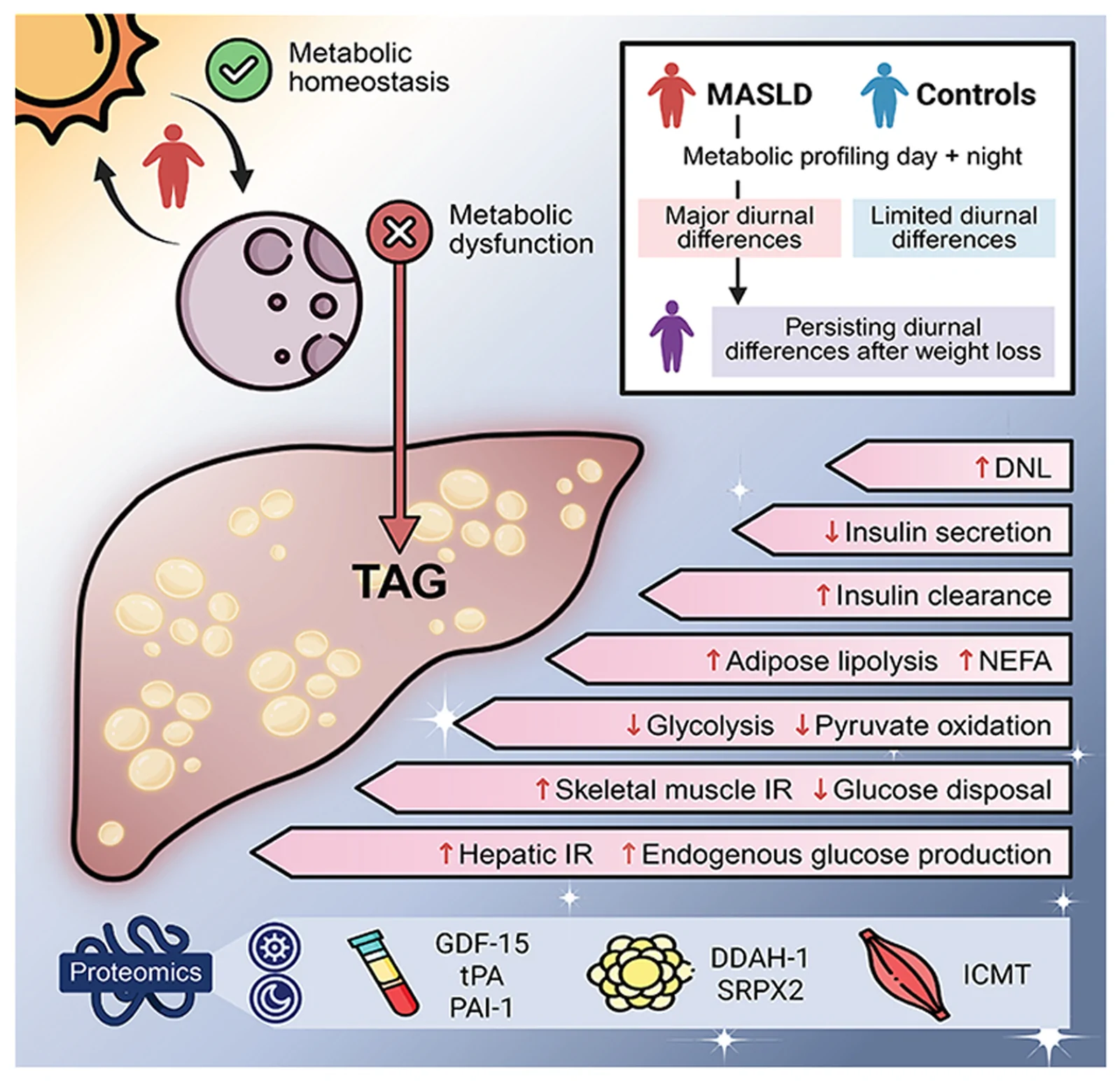

## Introduction

Metabolic dysfunction-associated steatotic liver disease (MASLD) affects approximately 40% of the global population and is closely associated with obesity and insulin resistance (IR).^1^ It is characterized by excess triacylglycerol (TAG) within hepatocytes, which can progress to inflammation (metabolic dysfunction-associated steatohepatitis [MASH]), cirrhosis, and hepatocellular carcinoma.^2^ MASLD is rapidly becoming the most common indication for liver transplantation worldwide^3^ and is responsible for a significant global burden of liver- and cardiovascular-related morbidity and mortality.^4^ Intrahepatic TAG is derived from esterification of fatty acids that reach the liver either from adipose tissue lipolysis, the diet, or through synthesis from non-lipid precursors during *de novo* lipogenesis (DNL).^5^ TAG is removed from the liver through β--oxidation or via secretion as a very low-density lipoprotein (VLDL). The pathogenesis of MASLD relies on an imbalance between these processes of hepatic lipid influx and synthesis vs. disposal.^6^

Multiple pre-clinical models have shown that liver metabolic homeostasis is strongly influenced by the circadian clock, an evolutionarily conserved mechanism synchronizing physiology and behavior across a 24-h cycle.^7^ Disruption of clock function in mice results in a range of adverse metabolic consequences including hepatic steatosis.^8,9^ In addition, gain and loss of body weight in mice has been shown to reprogram the circadian liver transcriptome, leading to a shift in the periodic abundance of circulating lipid metabolites.^10^ In healthy human volunteers, there also appear to be diurnal changes in glucose tolerance, being highest in the morning and reducing throughout the day.^11^ This is accounted for by variation in both insulin sensitivity and β cell responsiveness.^12–14^ However, despite hepatic metabolism and the circadian system being intrinsically linked, there have been no detailed assessments of diurnal glucose and lipid handling in patients with MASLD. Understanding tissue-specific daily metabolic rhythms in this patient group will therefore advance our understanding of disease pathogenesis and will inform practical approaches to harness or manipulate biological rhythms to improve metabolic and liver health. This includes establishing the optimal window for energy intake, exercise, and medication delivery (chronopharmacology) in patients with MASLD.

In the present study, we used an integrative physiological approach using state-of-the-art stable isotope techniques to characterize multiple metabolic pathways during day and night in patients with MASLD (before and after weight loss) as well as in overweight non-MASLD controls. Assessments of these functional pathways were complemented by high-throughput proteomic analysis of multiple sample types including plasma, adipose, and skeletal muscle tissue.

## Results

### Patients with MASLD have nighttime hepatic IR and increased EGP compared with controls

**Figure 1:**
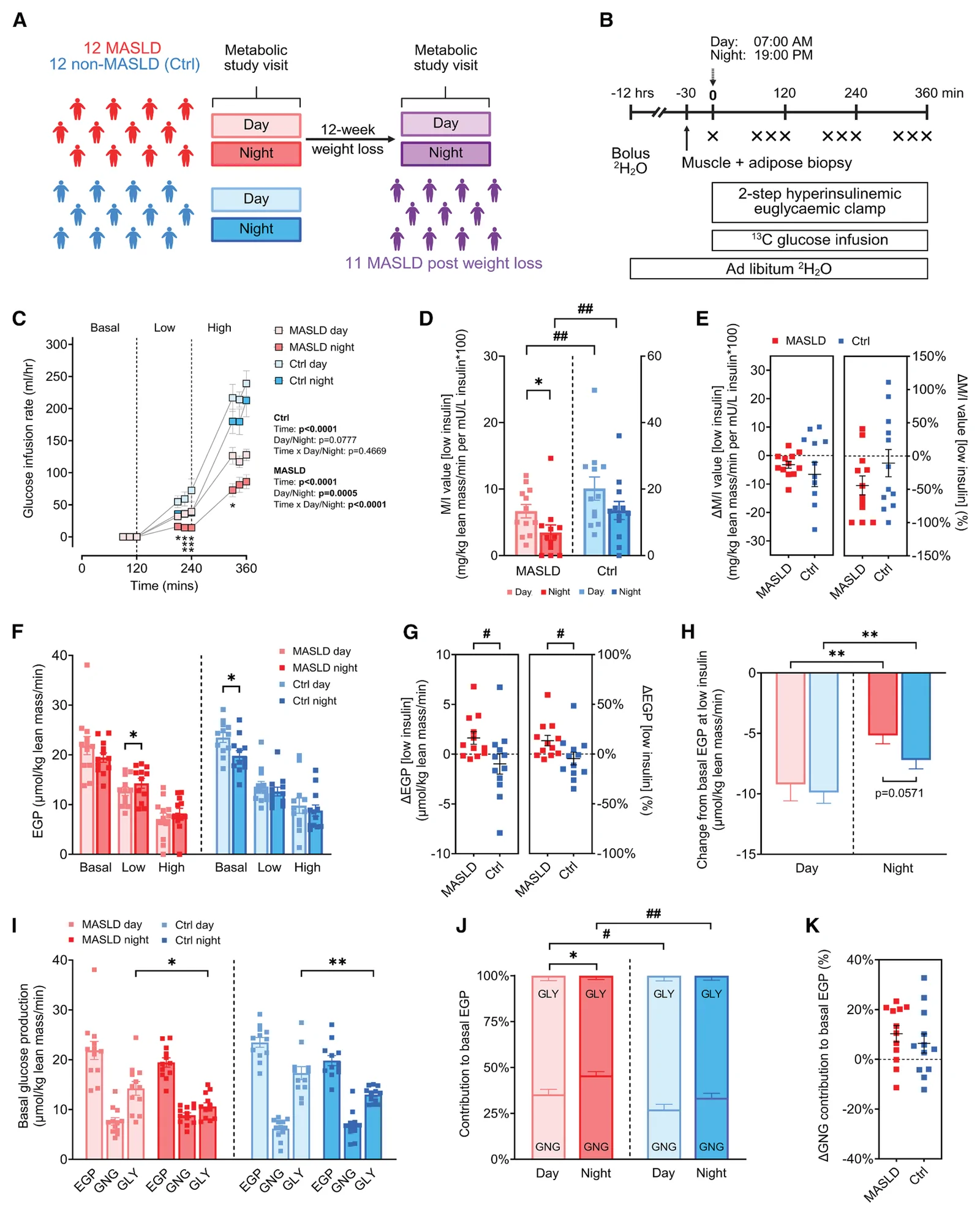
Patients with MASLD have exaggerated nighttime hepatic IR and elevated nighttime EGP (A) Study design. (B) Design of metabolic study visit. (C) Glucose infusion rates are required to maintain euglycemia across the two-step clamp. (D) Stable-state lean mass-adjusted M/I values at low-dose insulin. (E) Absolute and percentage change (Δ) (night-day) in M/I values at low-dose insulin. (F) Lean mass-adjusted EGP during basal, low-dose, and high-dose insulin. (G) Absolute and percentage change (Δ) (night-day) in EGP at low-dose insulin. (H) Low-dose insulin-mediated suppression of EGP. (l) Absolute contribution of GNG and GLY to basal EGP. (J) Percentage contribution of GNG and GLY to basal EGP. (K) Absolute change (Δ) in the percentage contribution of GNG to basal EGP (night-day). Data are represented as mean ± SEM. *n* = 12 in MASLD and 12 in Ctrl group. *p* values were determined by two-way ANOVA tests with Šidák multiple comparisons showing diurnal differences in MASLD (C); paired (✳) or unpaired (#) *t* tests with Welch correction (D–K). “X” in (B) denotes blood and breath sampling.

To investigate the effect of time of day on hepatic and peripheral insulin sensitivity, 12 patients with MASLD and 12 overweight volunteers without MASLD underwent a two-step hyperinsulinemic-euglycemic clamp during the day (07:00–13:00) and night (19:00–01:00) under standardized fasting conditions (Figures 1A and 1B). The order of daytime and nighttime assessments was randomly determined and separated by <2 weeks. All participants had equivalent calorie intake during the 7 days preceding each clamp, and the final meal consumed before attending each study visit was calorie-matched (Figure S1A). There was also a similar distribution in calorie intake across the day in both groups and before daytime and nighttime investigations (Figures S1B and S1C). Dietary composition was also equivalent for the last meal consumed and during 24 and 72 h prior to each study visit (Figures S2D–S2F). Participants had comparable levels of physical activity in the week preceding daytime and nighttime investigations and had identical sleep duration and mid-point of sleep the night before each study visit (Figures S2A–S2C). Patients with MASLD had a hepatic controlled attenuation parameter (CAP) value on transient elastography of ≥305 dB/m and controls had a value ≤288 dB/m. This increased the likelihood of groups having a high (>10%) and low (<5%) hepatic fat fraction, respectively.^15^ All participants were overweight (BMI > 25 kg/m^2^), non-diabetic, and non-cirrhotic. Clinical characteristics are presented in Table S1. Two sensitivity analyses were performed after removing a single young female (21 years) patient with MASLD (Table S2) and single female participant with active menstrual cycles (Table S3) in order to account for age and menstrual phase as potential circadian and metabolic confounders, respectively. These sub-analyses demonstrated no difference in any of the major diurnal outcomes reported. Within-group variance across key metabolic parameters are also reported in Table S4.

Stable plasma glucose concentrations were maintained throughout the two-step clamp in both groups; however, different glucose infusion rates were required to maintain euglycemia (fasting plasma glucose levels) between day and night in patients with MASLD (Figure 1C; Tables S5 and S6). This was associated with MASLD patients having a lower rate of glucose metabolism per unit of insulin (M/I value) at night, compared with day, during low-dose insulin, suggesting nocturnal hepatic IR (Figure 1D). In contrast, although controls were more insulin sensitive than patients with MASLD, there were no significant diurnal differences in M/I values at low insulin (Figure 1D). The absolute difference and percentage difference (Δ) in M/I values between night and day at low-dose insulin were equivalent between groups (Figure 1E).

In parallel with diminished nighttime M/I values, patients with MASLD also had a significant elevation in the rate of endogenous glucose production (EGP) at night, compared with day, at low insulin, which was not observed in controls (Figure 1F). This corresponded with a greater absolute and percentage diurnal change (night-day) in EGP in patients with MASLD, compared with controls (Figure 1G). In addition, the degree of insulin-mediated suppression of EGP at night was significantly diminished in patients with MASLD, compared with controls (Figure 1H). There were also differences in the relative contributions of gluconeogenesis (GNG) and glycogenolysis (GLY) to basal EGP according to group and time of day (Figure 1I; Table S7). Patients with MASLD had a higher proportion of GNG than controls during day and night, and the percentage contribution of GNG to EGP increased at night in patients with MASLD but not in controls (Figure 1J). The diurnal change in GNG contribution to basal EGP, however, was not significantly different between groups (Figure 1K).

### Patients with MASLD have greater peripheral (skeletal muscle) IR, compared with controls, but with similar diurnal differences observed in both groups

**Figure 2:**
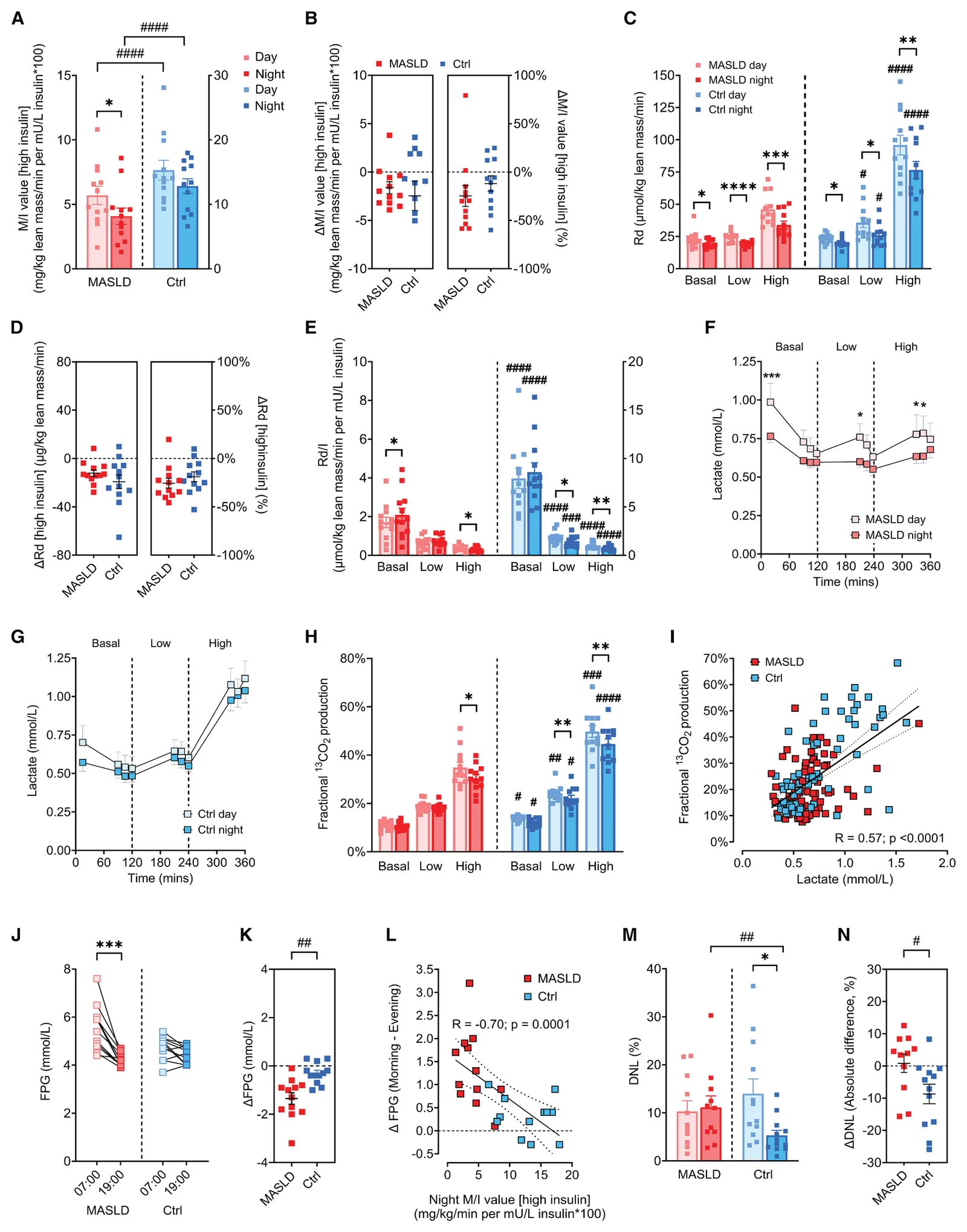
Patients with MASLD have exaggerated nighttime peripheral IR and upregulated nighttime DNL (A) Stable-state lean mass-adjusted M/I values at high-dose insulin in patients with MASLD and non-MASLD controls (Ctrl) during day and night. (B) Absolute and percentage change (Δ) (night-day) in M/I values at high-dose insulin. (C) Rate of lean mass-adjusted glucose disposal (Rd) during basal, low-insulin, and high-insulin phases of the two-step clamp. (D) Absolute and percentage change (Δ) (night-day) in Rd at high-dose insulin. (E) Rate of lean mass-adjusted glucose disposal corrected for plasma insulin concentration (Rd/I). (F) Plasma lactate concentration in patients with MASLD across the clamp during day and night. (G) Plasma lactate concentrations in Ctrl group across the clamp during day and night. (H) Percentage ^13^CO_2_ production in exhaled breath relative to the circulating ^13^C-glucose tracer pool, representing total-body complete pyruvate oxidation. (I) Linear regression between mean plasma lactate and exhaled ^13^CO_2_ percentage during stable state of all phases of the clamp. (J) Fasting plasma glucose (FPG) concentrations during morning and evening. (K) Absolute change (Δ) (evening-morning) in FPG. (L) Linear regression between nighttime M/I values at high-dose insulin and the degree of morning hyperglycemia (morning-evening FPG). (M) Proportion of basal DNL based on the incorporation of deuterium from ^2^H_2_O in plasma water into VLDL-TG palmitate. (N) Change (Δ) in the proportion of basal DNL (night-day). Data are represented as mean ± SEM. *n* = 12 in MASLD and 12 in Ctrl group (A–I); *n* = 11 in MASLD and 12 in Ctrl group (L and M) due to one MASLD patient failing to tolerate oral consumption of ^2^H^2^O. *p* values were determined by paired (✳) or unpaired (#) *t* tests with Welch correction (A–D, G, and I–M), two-way ANOVA tests with Šidák multiple comparisons (E and F), and linear regression (H and K).

Diurnal patterns of glucose metabolism were also observed during high-dose insulin; patients with MASLD had lower M/I values at night, compared with day, reflecting nocturnal peripheral IR (Figure 2A). Although control participants had higher M/I values than those with MASLD at both times of day, no significant diurnal differences were observed (Figure 2A). Nonetheless, the absolute difference and percentage difference (Δ) in M/I values between night and day at low-dose insulin were equivalent between groups (Figure 2B). Rates of lean mass-adjusted glucose disposal (Rd), reflecting peripheral (predominantly skeletal muscle) insulin sensitivity, were also reduced in patients with MASLD at night, compared with day, during low- and high-dose insulin conditions (Figure 2C). Similar diurnal patterns of Rd were observed in control participants who also had reduced nighttime values during basal, low-dose, and high-dose insulin phases of the clamp. This meant that the diurnal change observed remained equivalent between groups (Figure 2D). However, notably, Rd was reduced in patients with MASLD, compared with controls, across all clamp phases irrespective of time of day, indicating they are more insulin resistant (Figure 2C). Importantly, in the context of incomplete EGP suppression at high-dose insulin (Figure 1F), Rd remains a robust marker of insulin sensitivity whereas peripheral glucose uptake may be underestimated by M and M/I values. The diurnal differences in Rd also persisted after correcting for plasma insulin concentrations (Rd/I) (Figure 2E), indicating that diurnal patterns are related to peripheral insulin sensitivity and are not simply reflective of changes in plasma insulin concentrations (see below).

To try and interrogate glycolytic and pyruvate oxidation pathways, we analyzed plasma lactate concentrations and the proportion of exhaled ^13^CO_2_ (derived from infused ^13^C-glucose), respectively. In patients with MASLD, lactate tended to be lower at night, compared with day, but remained static across basal, low-, and high-insulin phases of the clamp (Figure 2F). In contrast, control participants had no significant diurnal differences, but there was an effect of clamp phase with lactate concentrations approximately doubling during high-dose insulin conditions (Figure 2G). Given that insulin stimulates the glycolytic action of phosphofructokinase (PFK) and promotes glucose uptake in adipose and skeletal muscle tissues, these data point toward systemic reductions in nighttime glycolysis in MASLD, likely resulting from IR. The enhanced rates of glycolysis in response to high-dose insulin observed in controls also reflect preservation of insulin sensitivity, compared with MASLD participants, and are consistent with previous clamp studies in healthy volunteers.^16^

Control participants also had a higher fractional ^13^CO_2_ production than patients with MASLD at low- and high-dose insulin irrespective of time of day (Figure 2H). However, both groups had a significant nighttime reduction in fractional ^13^CO_2_ production during high-dose insulin, reflecting impaired total-body insulin-mediated pyruvate oxidation via the pyruvate dehydrogenase complex (PDC). There was a strong correlation between plasma lactate and fractional ^13^CO_2_ production, consistent with glycolysis and pyruvate oxidation being connected pathways for intracellular glucose metabolism (Figure 2I).

### Overnight IR in patients with MASLD contributes to morning hyperglycemia and inappropriately maintained nighttime hepatic DNL

Due to the combination of overnight hepatic and peripheral IR, reduced glycolysis, and impaired pyruvate oxidation, patients with MASLD exhibited significant morning hyperglycemia (“dawn phenomenon”), compared with controls (Figures 2J and 2K). This is further exemplified by the negative correlation of morning hyperglycemia (relative to evening fasting plasma glucose) with nighttime M/I values during low- and high-dose insulin (Figure 2L). Morning hyperglycaemia in patients with MASLD was also mirrored by a higher morning Hep-IR index (fasting insulin × EGP), compared with evening values (493.6 ± 171.4 vs. 289.8 ± 77.9; *p* = 0.0005). Controls participants had similar diurnal patterns in Hep-IR (76.9 ± 6.2 vs. 52.9 ± 7.3; *p* = 0.0028), although values were significantly lower, compared with patients with MASLD, irrespective of time of day. The nighttime availability of substrate (glucose) alongside IR may also contribute to upregulated nighttime DNL in MASLD. Whereas controls had clear diurnal differences in DNL (being lower at night), the proportion of DNL remained elevated at night in patients with MASLD (Figure 2M). This resulted in a significant diurnal difference between groups (Figure 2M) and increased nighttime DNL in patients with MASLD, compared with controls (Figure 2N).

### Insulin fails to suppress adipose lipolysis at night in patients with MASLD, and this is incompletely offset by markers of β-oxidation and hepatic lipid export

**Figure 3:**
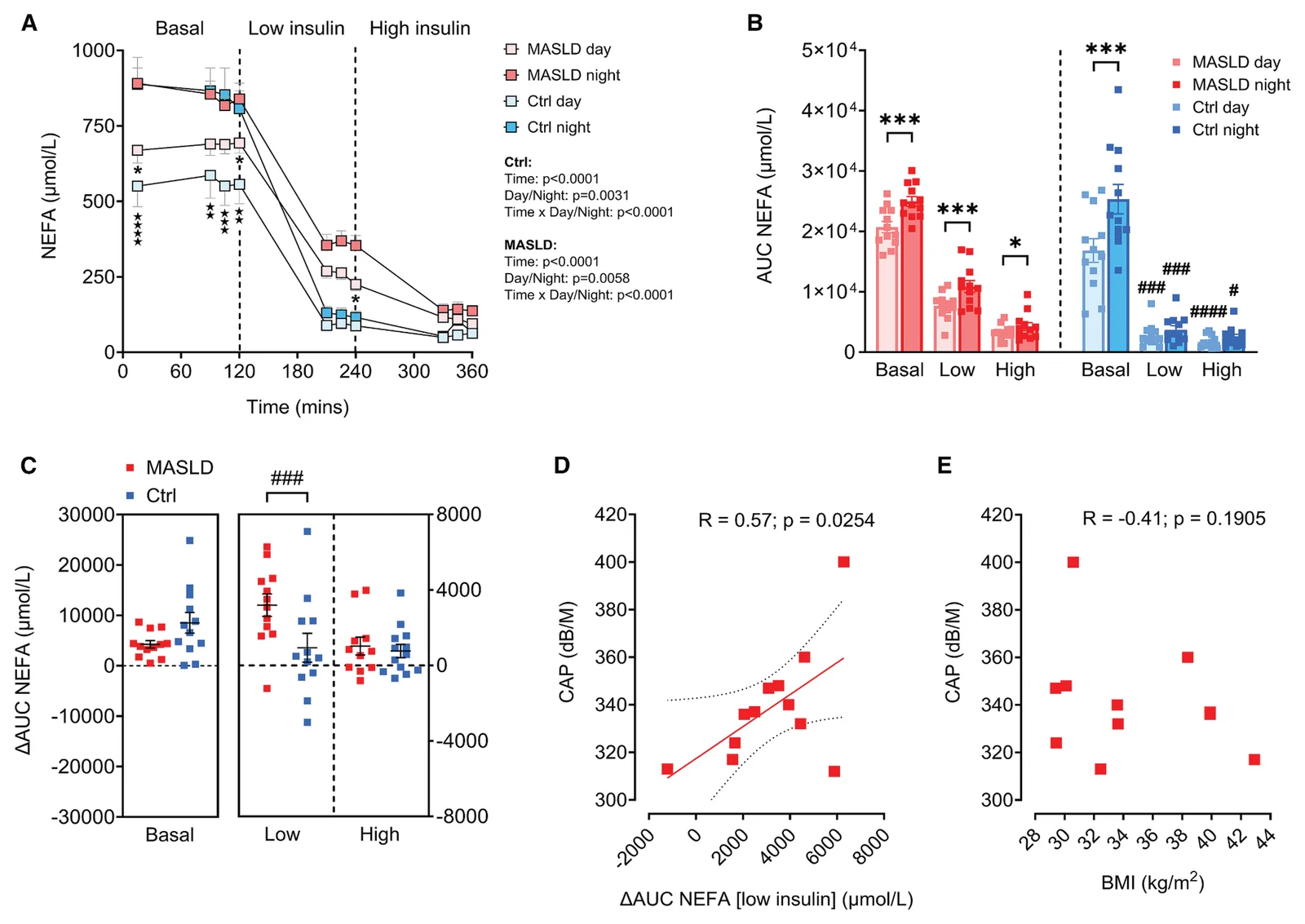
Patients with MASLD have elevated nighttime adipose lipolysis, which correlates with degree of hepatic steatosis (A) Trajectory of plasma NEFA across the two-step clamp during day and night in patients with MASLD and non-MASLD controls (Ctrl). (B) Area under the curve (AUC) for NEFA during basal, low-dose, and high-dose insulin phases of the two-step clamp. (C) Absolute change (Δ) (night-day) in AUC NEFA. (D) Linear regression between nighttime change in NEFA during low-dose insulin and CAP in patients with MASLD. (E) Linear regression between BMI and CAP in patients with MASLD. Data are represented as mean ± SEM. *n* = 12 in MASLD and 12 in Ctrl group. *p* values were determined by two-way ANOVA tests with Šidák multiple comparisons showing diurnal differences in MASLD (✳) and Ctrl (✶) (A), paired (✳) or unpaired (#) *t* tests with Welch correction (B and C), and linear regression (D and E).

Both groups showed nighttime elevations in basal non-esterified fatty acids (NEFAs) (Figures 3A and 3B). While insulin suppressed absolute NEFA concentrations to a similar extent in controls at both times of day, patients with MASLD had persistent elevations in nighttime NEFA during low- and high-dose insulin conditions (Figures 3A and 3B). In parallel with NEFA concentrations, identical diurnal patterns were observed with plasma glycerol, indicating a time-of-day effect on lipolysis (Figure S2). During low-dose insulin, the nighttime change in NEFA, compared with daytime, was significantly greater in patients with MASLD vs. controls (Figure 3C). These data suggest that patients with MASLD have unchecked lipolysis at night. While plasma NEFA levels are an imperfect marker of lipolysis, as they reflect both fatty acid release and disposal, the fact that concentrations strongly correlated with plasma glycerol (which is minimally taken up by peripheral tissues) supports the interpretation that NEFA levels predominantly reflect lipolytic activity (Figures S3C and S3D). In addition, the nocturnal elevation in NEFA during low-dose insulin correlated with the degree of hepatic steatosis measured by transient elastography (Figure 3D), and the strength of this correlation was greater than that observed between BMI and CAP (Figure 3E). To explore whether markers of lipolysis could be accounted for by differences in BMI between groups, correlation analysis was performed between fat mass (derived from dual-energy X-ray absorptiometry [DEXA]) and fasting NEFA (Figure S3E). This did not show any significant correlations in either group, irrespective of time of day, which is consistent with previous data showing that lipolysis is more reflective of IR/sensitivity than degree of adiposity.^17^

**Figure 4:**
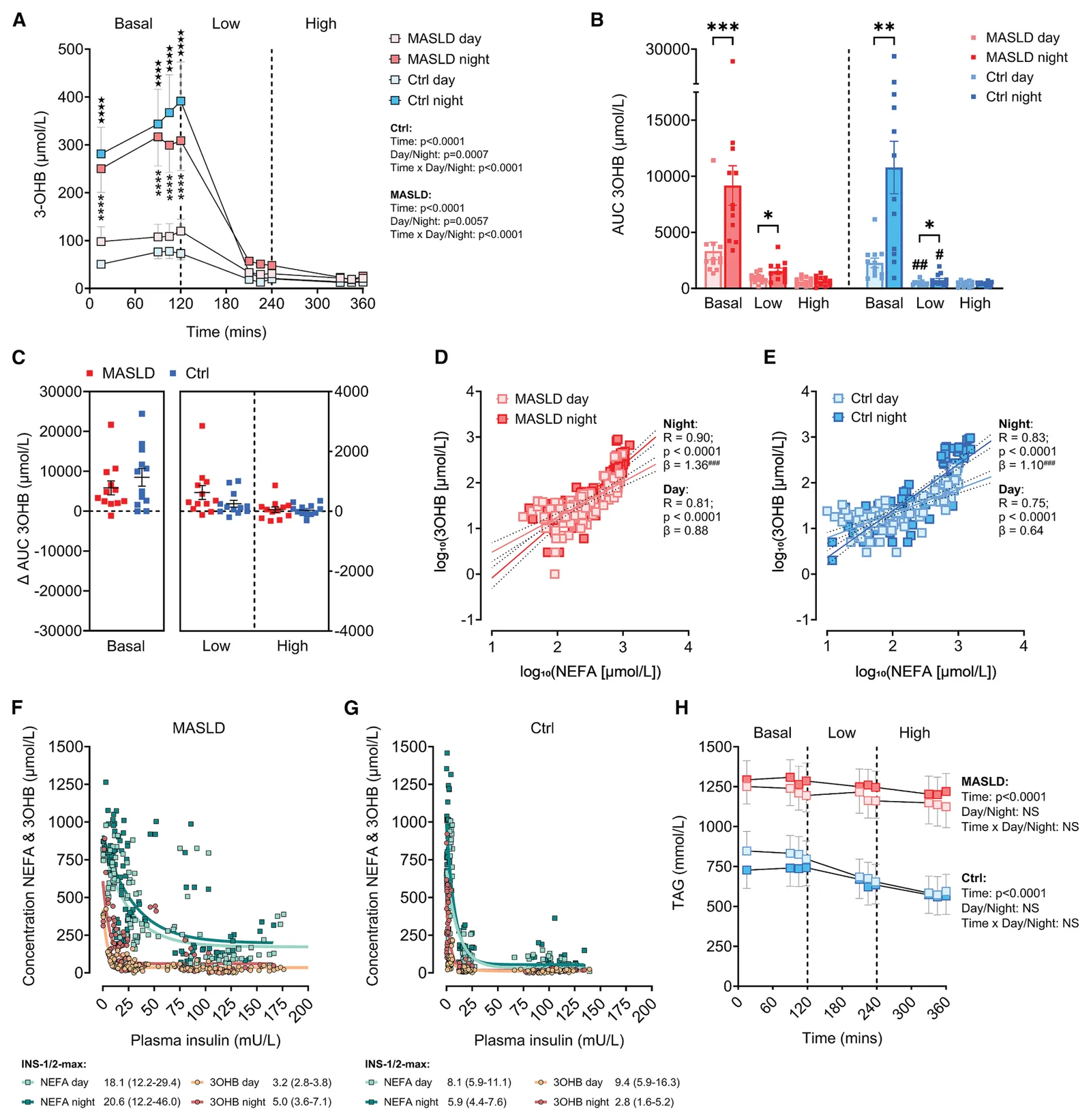
Increased nighttime adipose lipolysis is not offset by β-oxidation or hepatic TAG export (A) Trajectory of plasma 3OHB across the two-step clamp during day and night in patients with MASLD and non-MASLD controls (Ctrl). (B) AUC 3OHB. (C) Absolute change (Δ) (night-day) in AUC 3OHB. (D and E) Correlation and linear regression analysis between NEFA and corresponding 3OHB concentrations across x10 sampling time points (Figure 1B) for each participant split by day and night in patients with MASLD (D) and in Ctrl (E). (F and G) Scatterplot of NEFA and OHB concentrations with corresponding insulin concentrations across x10 clamp time points for each participant split by group and day/night in patients with MASLD (F) and in Ctrl (G). INS-1/2-max denotes the insulin concentration required to achieve 50% reduction in plasma 3OHB or NEFA. (H) Trajectory of plasma triacylglycerol (TAG) concentrations across the two-clamp. Data are represented as mean ± SEM. *n* = 12 in MASLD and 12 in Ctrl group. *p* values were determined by two-way ANOVA tests with Šidák multiple comparisons showing diurnal differences in MASLD (✳) and Ctrl (✶) (A and H), paired (✳) or unpaired (#) *t* tests with Welch corrections (B), and linear regression (D and E). Correlation analysis was performed using Spearman correlation coefficient and linear regression (D and E). INS-1/2-max values (F and G) were estimated using non-linear regression (one-phase exponential decay), assuming that INS-1/2-max = ln(2)/κ.

Mitochondrial β-oxidation of NEFA to ketone bodies is an important route of intrahepatic lipid disposal. We observed significant diurnal differences in basal levels of plasma 3-hydroxybutyrate (3OHB), as a product of β-oxidation, with much higher values observed at night, compared with day, in both groups (Figures 4A–4C). 3OHB production was subsequently suppressed by intravenous insulin in both groups, and no diurnal differences were observed during the high-dose insulin phase of the clamp. Plasma NEFA concentrations positively correlated with 3OHB during day and night in both groups (Figures 4D and 4E). However, both the strength of the correlation (R) and the slope of the linear regression (β) were significantly higher at nighttime compared with daytime.

Furthermore, in patients with MASLD, there were clear differences in the relative sensitivity of β-oxidation and lipolysis pathways to the inhibitory effects of insulin (Figure 4F). We showed a discrepancy between the concentration of insulin required to achieve 50% suppression of plasma 3OHB vs. NEFA. Indeed, five times more insulin was required at night to suppress lipolytic vs. β-oxidation pathways in MASLD, whereas inhibitory curves were well matched in the control group (Figure 4G). In addition to β-oxidation, the liver can also clear intrahepatic triglyceride through lipid export (predominantly as VLDL). During both day and night, patients with MASLD had higher plasma TAG concentrations than controls across all phases of the clamp (Figure 4H). Although there was a significant effect of time (across the clamp) on circulating TAG, there were no differences between day and night in either group. Taken together, these data suggest that high rates of nighttime lipolysis and resulting NEFA flux to the liver in patients with MASLD are incompletely offset by nocturnal upregulation of β-oxidation or hepatic lipid export.

### Nocturnal IR in patients with MASLD is compounded by reduced ISR and inappropriate insulin clearance at night

**Figure 5:**
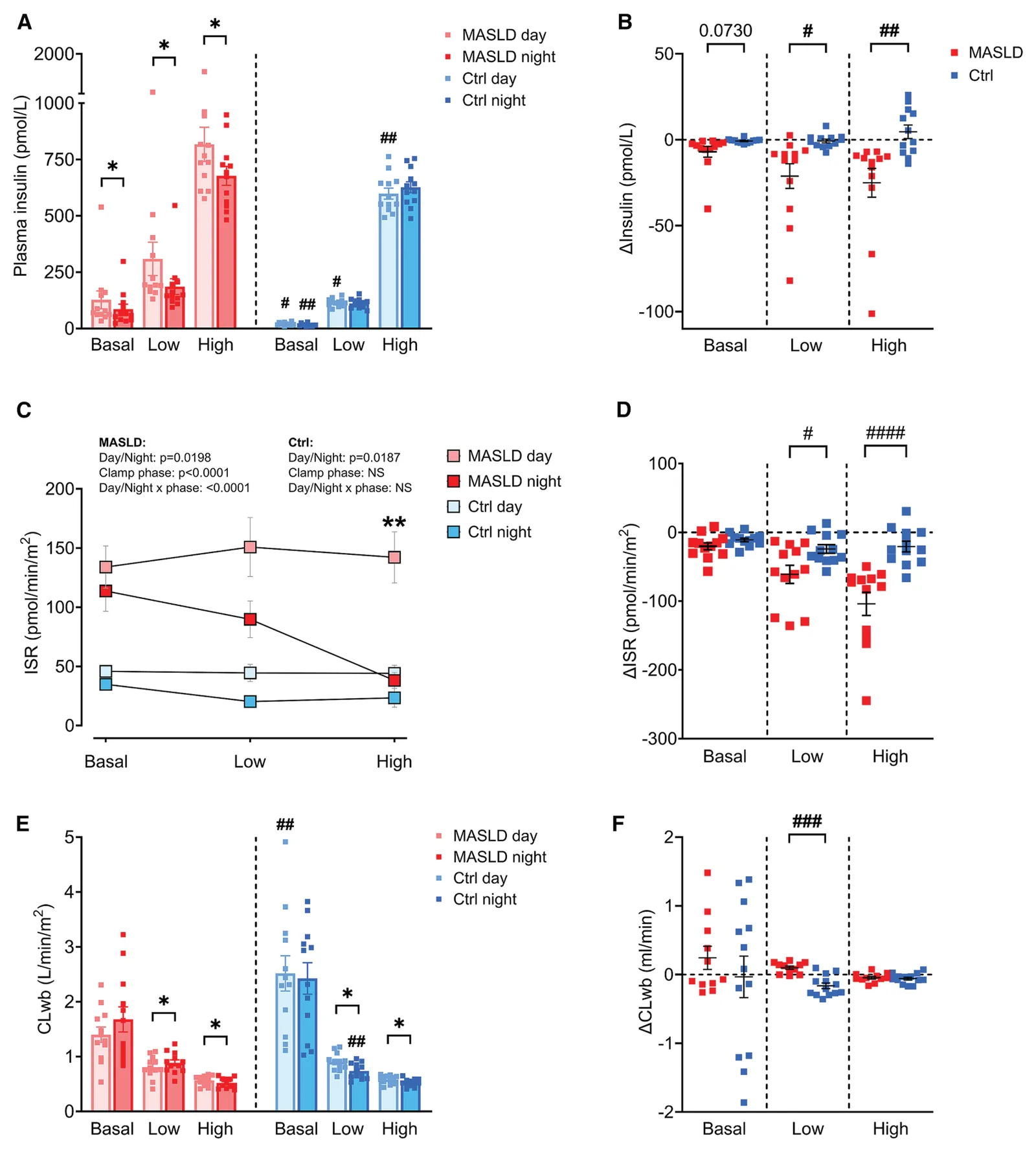
Nocturnal IR in patients with MASLD is compounded by reduced ISR and inappropriate insulin clearance at night (A) Plasma insulin concentrations during basal, low-dose, and high-dose insulin phases of the two-step clamp in patients with MASLD and in Ctrl. (B) Absolute change (Δ) (night-day) in plasma insulin. (C) ISR across basal, low-dose, and high-dose insulin phases of the two-step clamp according to group and day/night. (D) Absolute change (Δ) (night-day) in ISR. (E) CLwb. (F) Absolute change (Δ) (night-day) in CLwb. Data are represented as mean ± SEM. *n* = 12 in MASLD and 12 in Ctrl group. *p* values were determined by paired (✳) or unpaired (#) *t* tests with Welch correction (A, B, and D–F) and two-way ANOVA tests with Šidák multiple comparisons (C).

Across all stages of the clamp, we identified significant reductions in plasma insulin concentrations at night, compared with day, in patients with MASLD despite identical rates of infused insulin, whereas concentrations remained unchanged between day and night in controls (Figure 5A; Table S8). This resulted in a significant difference between groups in the absolute diurnal change (Δ) in plasma insulin during low- and high-dose insulin phases (Figure 5B). While patients with MASLD had hyperinsulinemia relative to controls during the day, the two groups had comparable insulin concentrations at night during low- and high-dose insulin (Figure 5A). To try and explain this MASLD-specific reduction in nighttime plasma insulin availability, we used a validated model integrating plasma insulin and C-peptide profiles (Figure S4) to estimate rates of endogenous insulin secretion (ISR) and whole-body insulin clearance (CLwb).^18^ Patients with MASLD had much higher basal state ISR, consistent with IR. However, patients with MASLD exhibited a dramatic (>66%) reduction in ISR, specifically at night, in response to insulin (Figure 5C). This effect was not observed in controls and therefore the diurnal change in ISR was significantly different between groups during low- and high-dose insulin (Figure 5D). Compounding this insulin-stimulated, reduced nighttime ISR, patients with MASLD also had inappropriate upregulation of insulin clearance at night. Basal CLwb was lower in MASLD, compared with controls, during the day but not at night (Figure 5E), and the diurnal change in CLwb during low insulin was significantly greater in MASLD compared with that in controls (Figure 5F). In both groups, there was a reduction in CLwb with increasing infusion rates of insulin, consistent with previous studies^19^ (Figure 5E).

### Plasma proteomics identifies diurnal expression of GDF-15 in patients with MASLD, which strongly associates with markers of β-oxidation

**Figure 6:**
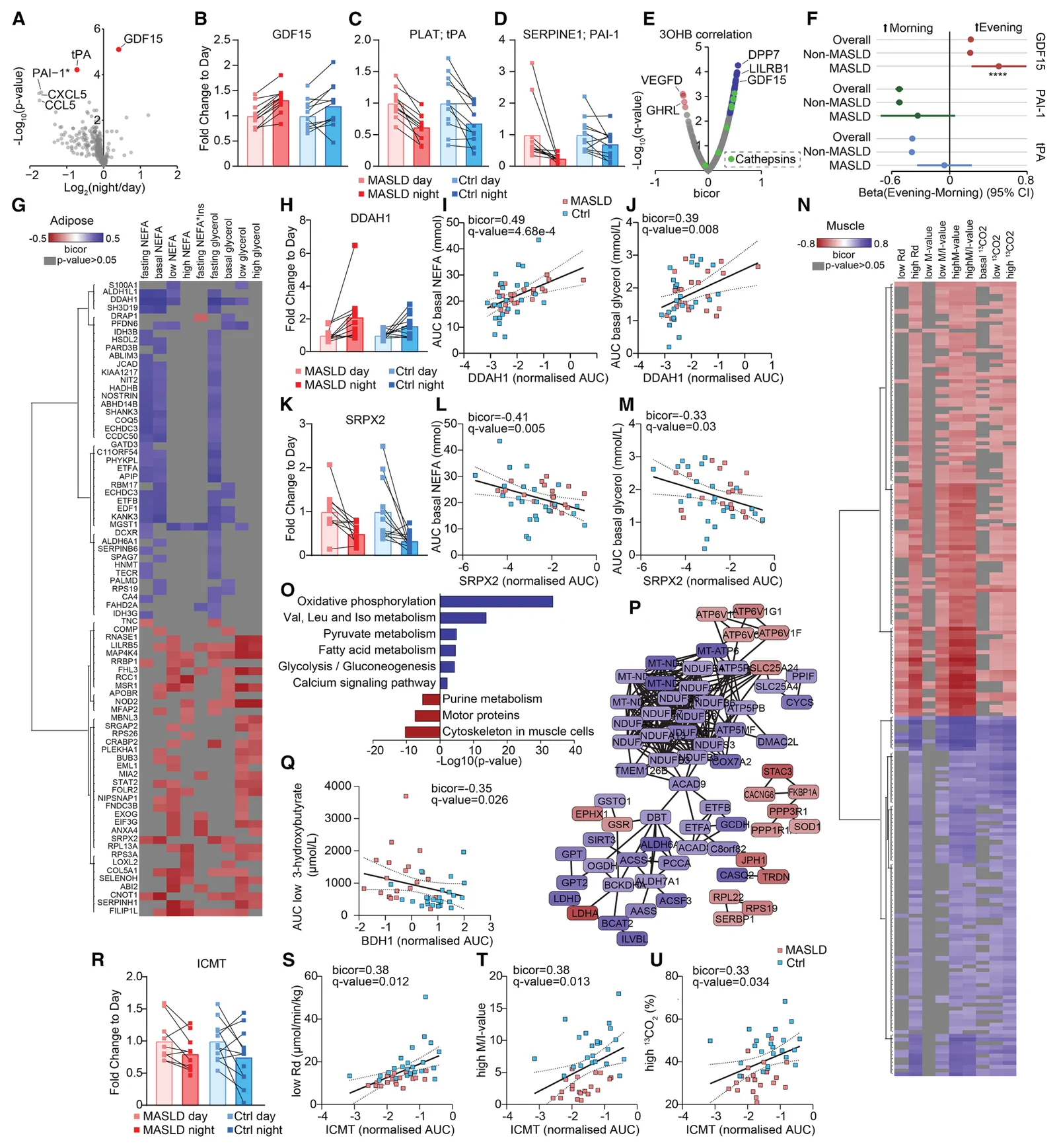
Plasma and adipose and skeletal muscle proteomics identifies diurnal associations to metabolic phenotypes in MASLD (A) Volcano plot showing differential expression (night vs. day) of plasma cardiometabolic proteins in patients with MASLD. (B–D) Relative quantification of indicated differentially expressed plasma proteins. (E) Correlation of individual plasma proteins and plasma 3OHB levels. (F) Forest plot showing differential expression of proteins (night vs. day) in UK Biobank adjusted for age, sex, ethnicity, Townsend deprivation index, assessment center, smoking status, alcohol intake, and BMI. (G) Heatmap of adipose tissue proteins correlated to plasma NEFA and glycerol concentrations across the two-step hyperinsulinemic clamp. (H) Relative quantification of DDAH1 in adipose tissue. (I and J) Correlation of adipose DDAH1 to basal plasma NEFA and glycerol levels. (K) Relative quantification of SRPX2 in adipose tissue. (L and M) Correlation of adipose SRPX2 to basal plasma NEFA and glycerol levels. (N) Heatmap of skeletal muscle proteins correlated to rates of glucose disposal (Rd), M/I values, and fractional ^13^CO_2_ production across the two-step hyper-insulinemic clamp. (O) KEGG pathway analysis of skeletal muscle proteins correlated to metabolic traits. (P) STRINGdb protein:protein interaction network of skeletal muscle proteins correlated to metabolic traits. (Q) Correlation of skeletal muscle BDH1 and plasma 3OHB levels. (R) Relative quantification of ICMT in skeletal muscle. (S–U) Correlation of skeletal muscle ICMT to glucose disposal, M/I value, and exhaled ^13^CO_2_ percentage. Data represent *n* = 12 in MASLD and 12 in Ctrl group (A–E and G–U). Data in (F) represent 9,493 non-MASLD and 121 MASLD for GDF-15 and PAI-1 and 9,468 non-MASLD and 121 MASLD for tPA. The significance threshold in (A) was set at *p* < 0.05 after adjusting for multiple comparisons; points below this threshold are colored red. ✳ in (A) denotes borderline statistical significance (adjusted *p* = 0.058). Bicor = biweight midcorrelation.

To determine whether functional metabolic readouts were associated with diurnal patterns of protein expression, we analyzed the proteome in plasma, adipose, and skeletal muscle (Figure S5A; Tables S9, S10, S11, S12, S13, S14, and S15). The Olink Explore proximity extension assay was used to quantify 691 cardiometabolic and inflammatory plasma proteins (Tables S14 and S15). In patients with MASLD, differential expression analysis of the plasma proteomics data identified growth differentiation factor 15 (GDF-15) and tissue-type plasminogen activator (tPA; PLAT) as significantly upregulated and downregulated at night compared with day, respectively (Figures 6A–6C). Plasminogen-activator inhibitor-1 (PAI-1; SERPINE1) was also downregulated at night-time, reaching borderline significance after adjustment for multiple comparisons (adjusted *p* = 0.058) (Figure 6D; Table S14). No significant diurnal differences were identified in controls (Table S14). Our intensive experimental protocol permitted only a modest sample size, which likely contributes to our identification of a limited number of proteins differentially regulated when segregating the groups and adjusting for multiple hypothesis testing with stringent false discovery rate (FDR). We therefore prioritized a correlation analysis of each individual protein to metabolic parameters in a pairwise manner to leverage variation in personalized responses. Here, we focused on proteins associated with plasma 3OHB given the large diurnal differences observed between the groups, particularly in the basal state when plasma samples for proteomic analysis were collected. This allowed us to identify 72 proteins positively and 4 proteins negatively correlated with 3OHB (Figure 6E; Table S11). The top positively correlated proteins include dipeptidyl peptidase 2 (DPP7), leukocyte immunoglobulin-like receptor subfamily B member 1 (LILRB1), and GDF-15. We also noted several lysosomal proteins present in plasma that correlated with plasma 3OHB, including cathepsins and enzymes involved in posttranslational processing of secretory proteins.

To validate the diurnally regulated proteins observed in our dataset, we analyzed antibody-based Olink plasma proteomic data from 54,219 individuals enrolled in the UK Biobank.^20^ The timing of plasma sampling in the UK Biobank was spread across the entire day (99.6% samples taken between 08:00 and 20:00 h) (Figure S5B) and therefore analysis was performed to evaluate differential protein expression between morning (before 10:00 h) (*n* = 2,894) and evening (after 18:00 h) (*n* = 6,943). This showed identical patterns to our clinical study with elevated GDF-15 in the evening and elevated tPA and PAI-1 in the morning (Figure 6F). Furthermore, patients with MASLD, identified by International Classification of Disease (ICD) coding (*n* = 124), demonstrated exaggerated diurnal changes in GDF-15, compared with controls, after adjusting for a range of covariates including age, sex, and BMI (*p* = 0.0001) (Figure 6F). However, the sample size of individuals with MASLD was small, and reliance on ICD-10 coding would have led to underestimation of true MASLD cases.

### Adipose and skeletal muscle proteomics demonstrates correlations with diurnal metabolic phenotype

Adipose and skeletal muscle biopsies were collected immediately prior to commencing the clamp at daytime and nighttime study visits, and liquid chromatography-tandem mass spectrometry (LC-MS/MS) was used to quantify 3,936 and 4,787 in the respective tissues (Tables S9 and S10). In patients with MASLD, only FCN2 was differentially abundant in adipose tissue according to day/night with borderline significance (adjusted *p* = 0.096), and no proteins were differentially abundant in skeletal muscle.

In controls, two adipose proteins (EMD and INF2) were significantly downregulated at night compared with day, while PPOX, UPE2M, and APOE were borderline significant after adjustment for multiple comparisons (adjusted *p* = 0.057, 0.069, and 0.061, respectively). No skeletal muscle proteins were differentially expressed in the control group. We reasoned that pooling data from MASLD and controls and performing a pairwise correlation analysis of proteomic data with metabolic parameters would provide greater power and leverage individual variation across the cohort. For adipose tissue, we focused on proteins associated with plasma NEFA and glycerol as markers of lipolysis. We identified 404 proteins that correlated with either NEFA or glycerol and 79 proteins that correlated with both metabolites either in the fasting state or at low- or high-dose insulin (Figure 6G; Table S12). This analysis identified proteins previously associated with lipid metabolism (e.g., HADHB and HSDL2) but also several novel candidates that displayed evidence of diurnal regulation. These included dimethylarginine dimethylaminohydrolase 1 (DDAH1), which was elevated at night and positively correlated with both NEFA and glycerol in both groups (Figures 6H–6J). We also identified a number of proteins with negative correlations, including in sushi repeat-containing protein (SRPX2), which were downregulated at night (Figures 6K and 6L).

For skeletal muscle, we focused on identifying proteins associated with markers of peripheral glucose metabolism including Rd glucose, M/I values, and fractional ^13^CO_2_ production at low or high insulin concentrations. We identified 202 proteins that were positively or negatively correlated with at least 2 of these phenotypic features (Figure 6N; Table S13). KEGG pathway enrichment analysis revealed positive associations between these proteins and oxidative phosphorylation, as well as metabolism of branched-chain amino acids, pyruvate, fatty acids, and glucose (Figure 6O). Negative associations were observed with pathways involved in purine metabolism and in motor and cytoskeletal protein synthesis (Figure 6O). We then used STRINGdb to map protein-protein interaction networks, integrating these pathway associations with rates of glucose disposal (Figure 6P). This analysis revealed a strong association between mitochondrial complex I and Rd glucose. The abundance of both hydroxybutyrate dehydrogenase (BDH1) and succinyl-coenzyme A (CoA):3-ketoacid CoA transferase 1 (OXCT1) in skeletal muscle was positively correlated to Rd glucose, M/I values, and fractional ^13^CO_2_ production. These two enzymes are the key rate-limiting steps in extra-hepatic ketolysis, and we found that BDH1 negatively correlated with plasma 3OHB (Figure 6Q). Protein-S-isoprenylcysteine o-methyltransferase (ICMT) was also positively correlated with all three markers of peripheral glucose handling and was downregulated at night (Figures 6R–6U).

### Diurnal metabolic differences persist after weight loss and liver fat reductions, suggesting that nighttime metabolic dysfunction is a primary driver of MASLD

Having established that diurnal metabolic rhythms are a feature of MASLD, we next aimed to determine whether nighttime metabolic dysfunction was more likely to represent a cause or an effect of hepatic steatosis. We therefore sought to “de-fat” the liver through a 12-week commercially available lifestyle and weight-loss program, after which identical follow-up (FU) daytime and nighttime study visits were completed (Figure 1A).

**Figure 7:**
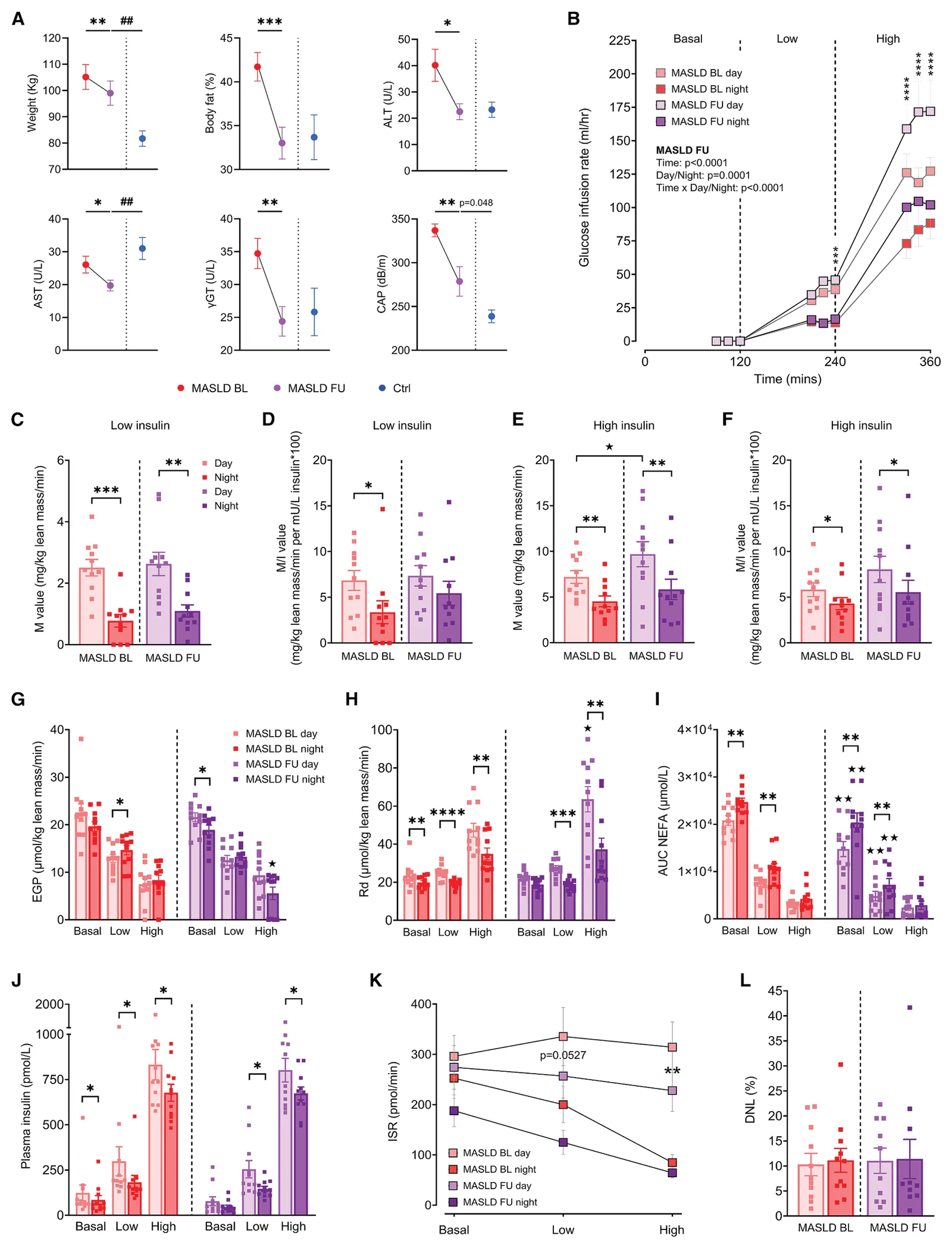
Diurnal metabolic differences persist after weight loss and liver fat reductions, suggesting that nighttime metabolic dysfunction is a primary driver of MASLD (A) Global improvements in markers of cardiometabolic and liver health in patients with MASLD after 12-week weight loss intervention. (B) Glucose infusion rates required to maintain euglycemia across the two-step hyperinsulinemic clamp in MASLD patients at BL and FU. (C) Lean mass-adjusted glucose metabolism (M values) at low-dose insulin. (D) Lean mass-adjusted glucose metabolism at low-dose insulin corrected for plasma insulin concentrations (M/I values). (E) Lean mass-adjusted glucose metabolism at high-dose insulin (M values). (F) Lean mass-adjusted glucose metabolism at high-dose insulin corrected for plasma insulin concentrations (M/I values). (G) Lean mass-adjusted EGP. (H) Rates of lean mass-adjusted glucose disposal (Rd). (I) AUC for plasma NEFA. (J) Plasma insulin concentrations. (K) ISR. (L) Proportion of DNL during basal phase of the clamp. Data are represented as mean ± SEM. Data represent *n* = 11 in MASLD BL, 12 in Ctrl, and 11 in MASLD FU group, unless otherwise stated below. *p* values were determined by paired (✳/✶) or unpaired (#) *t* tests with Welch correction (A, C–J, and L). ✶ in (H) and (I) represent significant *p* values for paired *t* tests between MASLD BL and MASLD FU for each corresponding time (day/night) and clamp phase. *p* values in (B) and (K) were determined by two-way ANOVA tests with Šidák multiple comparisons showing diurnal differences in MASLD FU only.

Patients with MASLD at FU had a 6.3% (±1.5%) body weight reduction alongside improvements in BMI, fat mass percentage, and liver biochemistry (Figure 7A; Table S1). Hepatic CAP values decreased to levels similar to those of the control group and fell below the threshold used to define a low liver fat fraction at enrollment (Figure 7A). Although weight loss was associated with improvements in metabolic health (Figure 7A), many of the diurnal differences observed at baseline (BL) persisted. This included preserved separation between day and night in the rates of infused glucose required to maintain euglycemia across the clamp, resulting in ongoing nighttime reductions in M and M/I values (Figures 7B–7F). Similar patterns in EGP to BL were also observed at FU, although the nighttime elevation in EGP at low-dose insulin did not reach statistical significance (Figure 7G). Nocturnal elevations in NEFA and reductions in Rd, plasma insulin, and ISR all persisted at FU (Figures 7H–7K), as did the constitutive upregulation of DNL at night (Figure 7L). The fact that these diurnal patterns were preserved despite a significant reduction in the degree of hepatic steatosis suggests that nighttime metabolic dysfunction may represent a primary driver of MASLD. Interestingly, the diurnal difference in the rate of Rd became even more pronounced as weight loss managed to improve daytime but not nighttime values (Figure 7H). This further reinforces the notion that the metabolic performance of skeletal muscle, specifically at night, may be a novel risk factor for MASLD.

## Discussion

We have shown that patients with MASLD have multiple pathways upregulated at night, which are known to contribute to intrahepatic TAG accumulation. These included significant diurnal differences, compared with controls, in EGP, lipolysis, DNL, ISR, and insulin clearance. The impact of nutritional challenges on circadian homeostasis has been well documented in mice. For example, temporal metabolomic analysis has demonstrated that high-fat diet (HFD)-induced obesity can lead to rewiring of the circadian clock, with both loss and gain of rhythmicity in gene expression with variable patterns observed across different tissue types.^10,21^ However, the relationship between metabolic stress and diurnal physiology in humans remains unknown. While dampened rhythms in circulating mediators, including adiponectin, leptin, cortisol, and FGF21, have been reported in obese human subjects,^22^ our data show that diurnal differences in functional metabolic pathways are in fact exaggerated in patients with MASLD. There are notable other clinical conditions where enhanced biological rhythms are associated with disease pathogenesis^23^ including asthma and rheumatoid arthritis.^24–27^ We suggest that MASLD should join these examples as a strikingly diurnal disease where exaggerations in normal circadian metabolic physiology ultimately become pathogenic.

The two-step hyperinsulinemic euglycaemiac clamp allowed us to report tissue-specific patterns of insulin sensitivity. Night-time hepatic IR was demonstrated by reduced nighttime M/I values and elevated rates of EGP under low-dose insulin conditions. A further original finding of our study is that the percentage contribution of GNG to EGP significantly increased at night in patients with MASLD but not in controls. This aligns with previous isotope studies showing that GNG represents the main contributor to EGP in patients with MASLD^28^ and is positively associated with the degree of hepatic inflammation and fibrosis.^29^ However, it is worth noting that our GNG values tended to be lower than those of a previous work, which may relate to the quantification methods used or variability in the timing of heavy water dosing.^28,30,31^ While we utilized well-established protocols for measuring hepatic glucose flux *in vivo*,^32–34^ we recognize that this derivatization approach may overestimate fractional GNG at lower values and underestimate it at higher values.^31^ The nighttime upregulation of GNG in our study supports work from Unni et al. who showed that overnight GNG is a major driver of morning hyperglycemia in patients with type 2 diabetes (T2D).^35^ Unni et al. and others have also demonstrated that the degree of carbohydrate consumption during the preceding >3 days can influence rates of GNG.^35,36^ It is therefore reassuring that participants in our study had equivalent dietary compositions during the same time frame before daytime and nighttime investigations. The diurnal variability of GNG may reflect shunting of excess nighttime glycerol into glucose production or be due to increased acetyl-CoA-mediated activation of pyruvate carboxylase by NEFA oxidation.^37,38^ Pre-clinical models have shown that gluconeogenic enzymes can become newly circadian in the context of weight gain and hepatic steatosis^10^; however, work in human MASLD has shown that GNG appears to be mostly governed by substrate availability rather than transcriptional changes in the liver.^29^

Excess glucose production in patients with MASLD was combined with striking nighttime reductions in Rd, representing impaired metabolic function of skeletal muscle. Reduced Rd is recognized as an important characteristic of MASLD, with previous clamp studies showing a 30% and 40% reduction, compared with controls, at low- and high-dose insulin, respectively.^39^ Similar findings are replicated in the current study, with patients with MASLD having 40%–60% reductions in Rd relative to the control group, irrespective of time of day. Although the diurnal change in Rd appeared to be of similar magnitude in both groups, low daytime Rd values in MASLD patients are likely to already fall below the threshold necessary to promote hyperglycemia, which is then exacerbated by further incremental nighttime reductions.

We show that nighttime elevations in plasma NEFA strongly correlated with hepatic steatosis as measured by CAP. This suggests that the cumulative burden of nighttime hepatic NEFA exposure over time may represent a novel risk factor for the development of steatotic liver disease, particularly since most of the intrahepatic lipid content in MASLD is known to be derived from circulating plasma NEFA.^5^ To some extent, this NEFA influx to the liver may be offset by upregulated β-oxidation, and we observed significant elevations in 3OHB at night compare with day in both groups. Furthermore, compared with day, there was a greater incremental change in plasma 3OHB per unit increase in plasma NEFA. This suggests that enhanced nighttime ketogenesis cannot be entirely explained by diurnal substrate availability. Our plasma proteomic analysis showed a significant nighttime upregulation of GDF-15, compared with daytime, in patients with MASLD. GDF-15 is a well-recognized marker of nutritional stress^40^ and has been shown to increase according to the stage of hepatic inflammation and fibrosis in patients with MASH.^41^ Elevated GDF-15 in mice has also been proposed as a possible protective mechanism in MASLD by promoting compensatory upregulation of hepatic β-oxidation.^42,43^ We also observed a clear positive association between GDF-15 levels and ketone body production to corroborate this hypothesis. It is also notable that a number of lysosome-related proteins in plasma positively correlated with 3OHB. Lysosomes have a key role in shuttling lipids to the mitochondria for β-oxidation, and lysosomal activity has been shown to be both clock-gated via BMAL1^44,45^ and to have a role in intrahepatic triglyceride accumulation.^46^

The two other plasma proteins identified were tPA and its inhibitor PAI-1, which help fine-tune intravascular thrombosis and fibrinolysis.^47^ These were both found to be differentially upregulated at 07:00 h, and although they are unlikely to explain the diurnal metabolic changes observed, they are noteworthy for the fact that similar daily rhythms have been postulated as a cause for the morning preponderance of myocardial infarction.^48–50^ Patients with MASLD are already at increased risk of cardiovascular disease; therefore, characterizing whether diurnal patterns of hemostasis can further predispose to morning cardiac events in this cohort is worth further investigation.

Exploratory analysis using untargeted proteomics in adipose and skeletal muscle tissue uncovered a number of regulators that may account for diurnal metabolic phenotype and may offer insights into the pathogenesis of MASLD. DDAH1 is the major enzyme responsible for clearance of asymmetric dimethylarginine (ADMA), an endogenous inhibitor of nitric oxide synthase. We found that DDHA1 was upregulated at night and positively correlated with markers of lipolysis. This contrasts with rodent data where augmented DDAH1 has been shown to improve metabolic phenotype and enzymatic activity is associated with reduced plasma NEFA concentrations.^51^ In this regard, upregulated nighttime DDAH1 in the current study may be a compensatory mechanism for nocturnal adipose dysfunction, and indeed DDAH1 has been shown to increase in response to a range of insults including congestive heart failure^52^ and ischemic stroke.^53^ Adipose SRPX2 was shown to be downregulated at night and negatively correlated with lipolytic markers in the current study. While little is known about the role of this protein in adipose biology, SRPX2 transcripts are reduced in subcutaneous adipose tissue of individuals with constitutionally low BMI.^54^ In skeletal muscle, proteomic network analysis highlighted mitochondrial complex I as a key regulator of glucose disposal. Clock gene-dependent acetylation of complex I *in vitro* has previously been shown to orchestrate rhythmic activity of mitochondrial oxidative phosphorylation.^55^ Further work is required to determine if the effect of diurnal mitochondrial function on peripheral glucose metabolism *in vivo*, particularly as insulin sensitizers, including metformin and thiazolidinediones, is able to exert their anti-diabetic effects through complex I inhibition.^56^ Lastly, we focused on individual skeletal muscle proteins with strong associations with metabolic phenotype, including BDH1 and IMCT. BDH1 plays a crucial role in both hepatic ketogenesis and extra-hepatic ketolysis. While hepatocyte Bdh1 overexpression can ameliorate liver injury in rodent MASLD,^57^ the role of skeletal muscle BDH1 in metabolic disease remains ill-defined. ICMT, which down trended at night, catalyzes the posttranslational modification of G proteins containing a CAAX motif. This includes Rac1, which is activated by ICMT during glucose-stimulated ISR.^58^ Rac1 is also known to govern insulin-mediated GLUT4 translocation and glucose uptake by skeletal muscle, offering a putative mechanistic link between ICMT and peripheral glucose disposal.^59^

An unanticipated finding in our study was the striking diurnal change in rates of ISR and clearance observed specifically in patients with MASLD. This resulted in a significant nighttime reduction in insulin availability, which acts as a second pathogenic “hit” on top of widespread IR. Our reported daytime parameters are consistent with the wider literature showing that MASLD is associated with relative hyperinsulinemia, elevated ISR, and reduced insulin clearance, compared with control cohorts.^60,61^ This aligns with our fundamental understanding of the pathogenesis of impaired glucose tolerance^60,62^ where in the context of IR, hyperinsulinemia develops in order to compensate for diminished insulin signaling. However, these insights have been gleaned exclusively from studies performed during the day. Our diurnal investigations suggest that an entirely separate paradigm exists at night whereby insulin secretory capacity becomes acutely compromised and insulin clearance inappropriately upregulated. The reason for such a profound reduction in ISR at night remains unclear. There is some evidence that NEFA can directly impair β cell function^63,64^; however, nighttime elevations in NEFA are unlikely to be entirely responsible given that ISR reaches a nadir during high-dose insulin conditions when NEFA is maximally suppressed. It has previously been shown in rodent islets that hyperglycemia can alter glycolytic and pyruvate oxidation pathways with subsequent knock-on effects on β cell mitochondrial function.^65^ It is plausible that the nighttime deficits in glycolysis and pyruvate oxidation observed in the current study may contribute to impaired ISR overnight. Plasma glucose was also significantly lower in MASLD during the evening, both in fasting state and during the clamp. This reduction in glycemia could further contribute to the reduced stimulus for ISR. Alternatively, previous studies have shown that insulin can suppress its own secretion *in vivo*.^66,67^ As a result, the observed reduction in ISR at night in patients with MASLD may be related to altered β cell sensitivity to negative feedback signals from infused intravenous insulin.

Regarding inappropriate insulin clearance, a distinct subpopulation of patients with T2D and elevated insulin clearance has been described. Sugiyama et al. prospectively performed a single-step hyperinsulinemic euglycemic clamp on 101 consecutive Japanese patients with newly diagnosed T2D. Of these, 44% had elevated insulin clearance arbitrarily defined as a metabolic clearance rate (MCR) of >700 mL/min/m^2^. MCR is a relatively crude measurement and does not account for the dynamics of ISR, peripheral clearance, and hepatic insulin extraction. Nonetheless, the authors proposed that a sizable subgroup of patients with T2D may have inappropriate insulin clearance as the principal driver of abnormal glucose metabolism.^68^ However, the reason for such marked diurnal variability in the current study remains unclear. Insulin clearance is known to be closely linked to the circadian clock, with loss of *Per2* increasing plasma insulin levels in mice through impaired insulin clearance.^69^ It is also plausible that steatotic liver disease may lead to new or increased amplitude of oscillations in genes involved in the hepatic insulin clearance pathway.^10^

Lastly, we show that nighttime metabolic dysfunction persisted in patients with MASLD despite 6.5% weight loss and associated liver fat reductions. This suggests that nocturnal phenotype may be a preexisting risk factor for steatosis, analogous to how nighttime hypertension can predict incident cardiovascular disease.^70^ However, we acknowledge that patients with MASLD continued to have a higher BMI than controls even after lifestyle intervention, making it challenging to fully isolate the contribution of steatosis to diurnal metabolism beyond generalized adiposity. Further weight loss, in excess of the 6.5% achieved, would likely require pharmacological or surgical intervention,^71,72^ which may have affected circadian physiology independently of BMI.^73,74^ Investigating patients with lean MASLD and IR would therefore be an interesting area of further study.^39^ However, this group accounts for a small proportion of the global disease burden, and exploring the effects of nutritional challenge (e.g., weight loss/gain) on diurnal metabolic rhythms would be logistically and ethically challenging.

Our findings indicate that specifically targeting nighttime metabolic dysfunction may be a promising treatment strategy for MASLD. A number of drug agents have been trialed in the context of MASLD, which are directed toward multiple pathways described in this work; however, the optimal timing of drug delivery to ensure maximal pharmacological action at night has not yet been investigated.^75^ Similarly, whether evening exercise in patients with MASLD can improve glycemic control by leveling out diurnal troughs in nighttime glucose disposal remains an interesting area of further investigation. A time-dependent effect of exercise has already been reported, with several randomized trials indicating that exercise completed in the evening may have a greater metabolic benefit, compared with morning.^76,77^ This may be a particularly beneficial avenue to pursue in MASLD given that low nighttime Rd remains fixed despite weight loss. In addition, the overnight IR reported in MASLD provides a strong rationale for these patients to avoid large evening calorie loads. This is supported by a range of observational data showing that distributing energy intake away from the end of the day is associated with improved metabolic health.^78^ It is therefore notable that the largest single meal in our cohort was consumed for dinner (40% of total daily calories), which is likely to constitute a major nutritional stress in MASLD patients when combined with nighttime metabolic dysfunction. Lastly, our work reinforces the importance of accounting for time of day when completing clinical and experimental metabolic research.^79^

### Limitations of the study

First, both daytime and nighttime investigations were performed following a 12-h fasting period. While this ensured identical experimental conditions and removed the confounding effect of diet, the act of fasting (particularly during the day) does not replicate the typical real-world experience. It also means that the diurnal metabolic changes reported cannot be fully extrapolated to post-prandial responses, which others have also shown to vary according to time of day.^13,80^ Indeed, it is important to recognize that while our study reported on insulin sensitivity, using a hyperinsulinemic euglycemic clamp, it did not employ dynamic assessments of glucose tolerance. Furthermore, peripherally infused insulin reaches the liver only via the hepatic artery, whereas endogenous insulin enters directly through the portal vein.^62^ As a result, hepatic insulin exposure is reduced during the clamp, potentially blunting suppression of EGP and lowering insulin clearance, compared with physiological conditions. Second, although all patients had an established clinical diagnosis of non-cirrhotic MASLD and had a high liver fat content confirmed by transient elastography, there was no liver biopsy requirement for enrollment and therefore the precise impact of hepatic inflammation and fibrosis stage on diurnal metabolism remains unknown. We also acknowledge the ability of CAP to quantify intrahepatic lipid decreases as steatosis progresses.^81^

Although all control participants were overweight, they did have a significantly lower BMI, compared with MASLD participants. Identifying BMI-matched controls without hepatic steatosis remains an inherent challenge within clinical MASLD research. Lastly, while our modest sample size was adequate to detect key diurnal and between-group differences, non-significant findings for some outcomes may reflect limited power rather than true equivalence. Statistical comparisons between MASLD and controls may also be influenced by within-group variability observed between daytime and nighttime measures.

In conclusion, by adopting an integrative physiological approach, we have been able to describe daytime and nighttime differences in multiple pathogenic pathways implicated in the development and progression of MASLD. Principally, we report that MASLD is a nocturnal disease, characterized by adverse metabolic features occurring at night across multiple organ systems, including adipose tissue, skeletal muscle, and the liver. This appears to be driven by a combination of diurnal changes in both insulin sensitivity, ISR, and insulin clearance.

## Star✶Methods

### Key Resources Table

**Table:** 

REAGENT or RESOURCE	SOURCE	IDENTIFIER
Biological samples		
Human plasma from MASLD patients and Ctrls	Clinical study in this paper sponsored by University of Oxford	Research ethics committee (REC) reference: 21/WS/0017
Human adipose tissue samples from MASLD patients and Ctrls	Clinical study in this paper sponsored by University of Oxford	REC reference: 21/WS/0017
Human skeletal muscle tissue samples from MASLD patients and Ctrls	Clinical study in this paper sponsored by University of Oxford	REC reference: 21/WS/0017
Chemicals, peptides, and recombinant proteins		
D-GLUCOSE (U-13C6, 99%) Microbiological/Pyrogen Tested	CK Isotopes Limited	CLM-1396-MPT
2.5LT Chloroform, for HPLC, stabilised with amylene	Fisher Scientific UK	10615492
2.5LT Methanol, extra pure, SLR	Fisher Scientific UK	10214490
2.5LT Hexanes, Certified AR for analysis, 95% Hexane	Fisher Scientific UK	10783601
2.5LT Cyclohexane, Certified AR for analysis, C6 H12	Fisher Scientific UK	10253470
2.5LT Acetone, Certified AR for analysis, meets analytical specification of Ph.Eur, C3 H6 O	Fisher Scientific UK	10162180
2.5LT Diethyl ether, >= 99.5%, Analytical reagent grade	Fisher Scientific UK	10785901
2.5LT Toluene, Certified AR for analysis C7H8	Fisher Scientific UK	10356390
Ethyl acetate AnalaR NORMAPUR ACS/R.PE Glass bottle 1L	VWR International Ltd	23882.3
2.5LT Ethanol absolute, for HPLC, C2 H6 O	Fisher Scientific UK	10428671
500ML Decane, 99+%, pure	Fisher Scientific UK	10497480
1-PENTANOL, 99+%, A.C.S. REAGENT	Merck Life Science UK Limited	398268 –1L
500GR Potassium hydrogen carbonate, SpeciFied, meets Ph.Eur., BP CHKO3	Fisher Scientific UK Ltd	10724001
1KG POTASSIUM CARBONATE FOR ANALYSIS EMSURE	Merck Life Science UK Limited	1.05E+09
Sodium bromide pure 500g	Scientific Laboratory Supplies Limited (SLS)	CHE3282
BIS(TRIMETHYLSILYL) TRIFLUOROACETAMIDE*(B	Merck Life Science UK Limited	T6381-25G
PYRIDINE, ANHYDROUS, 99.8%	Merck Life Science UK Limited	270970 –100ML
1LT Sulfuric acid min 95% d=1.83, SLR, extra pure, H2 O4 S	Fisher Scientific UK Ltd	10754001
PHENYLMETHANESULFONYL FLUORIDE SOLUTION	Merck Life Science UK Limited	93482 –50ML-F
Fatty acid methyl ester Std #21 50mg neat (7 components, 2% –41% by weight)	Thames Restek	RE35027
F.A.M.E. MIX, C4-C24, 100MG NEAT	Merck Life Science UK Limited	18919 –1AMP
Tripentadecanoin (C15:0)	Merck Life Science UK Limited	T4257-1G
Heptadecanoic Acid	Merck Life Science UK Limited	H3500-5G
17:0 PC (1,2-diheptadecanoyl-sn-glycero- 3-phosphocholine) Molecular Weight: 762.09	Merck Life Science UK Limited	850360P-200MG
Cholesteryl Heptadecanoate	Cambridge Bioscience	CAY21750-100mg
Methyl Tricosanoate, Standard for GC	Merck Life Science UK Limited	91478 –1G
Critical commercial assays		
Cardiometabolic and inflammatory panel proteomics	Olink	https://olink.com/products/olink-explore-3072-384
Insulin ELISA	Mercodia	10-1113-01
C-Peptide ELISA	Mercodia	10-1136-01
Deposited data		
Proteomic analysis of three sample types (plasma, muscle, adipose) according to group and time-of-day	This paper + Deposited in the ProteomeXchange Consortium	PXD061275
Data S1	This paper	Data S1 – Unprocessed source data underlying all graphs. Related to Figures 1, 2, 3, 4, 5, 7, and S1–S5
Software and algorithms		
R version 4.1.1	R Development Core Team, 2016	https://www.R-project.org/
Perseus	Tyanova et al.^82^	maxquant.net/perseus/
SPSS 25 software	IBM SPSS Statistics	https://www.ibm.com/support/pages/downloading-ibm-spss-statistics-25
GraphPad Prism	N/A	https://www.graphpad.com/features
Other		
Human plasma proteomic data from UK Biobank (Olink Explore 3072 Proximity Extension Assay)	UK biobank	REC reference: 11/NW/0382

### Experimental Model and Study Participant Details

#### Study protocol

We performed an experimental clinical study in human participants conducted within the Clinical Research Unit (CRU) of the Oxford Centre for Diabetes, Endocrinology and Metabolism (OCDEM) (Oxford, UK) between March 2020 and April 2024. This involved metabolic phenotyping of overweight patients with MASLD and controls without MASLD during the day and night. The primary outcome was diurnal change in hepatic de novo lipogenesis (DNL). Secondary outcomes included glucose utilization across a two-step hyperinsulinemic euglycemic clamp, endogenous glucose production, rate of glucose disposal, total body glucose oxidation, and changes in circulating metabolites including non-esterified fatty acids (NEFA), glycerol, 3-hydroxybutyrate (3OHB), and lactate. Rates of gluconeogenesis and the differential abundance of proteins in plasma, adipose, skeletal muscle were investigated as exploratory outcomes. After completing baseline day and night assessments, control participants ended their involvement in the study, whereas patients with MASLD received a 12-week lifestyle and weight loss intervention after which identical day and night-time investigations were performed. The clinical protocol received full ethical approval from the West of Scotland Research Ethics Committee (reference 21/WS/0017). The clinical study was registered at clinicaltrials.gov (NCT05962099). The study was conducted in accordance with the Declaration of Helsinki and each participant provided written informed consent after being explained the nature and potential risks of the studies. The study and manuscript were completed in line with strengthening the reporting of observational studies in epidemiology (STROBE) guidance (Document S2).

#### Recruitment and participant details

We recruited a total of 24 participants (12 patients with MASLD and 12 participants without MASLD). Patients had an established clinical diagnosis of MASLD and were recruited from metabolic hepatology clinics at Oxford University Hospitals NHS Foundation Trust. Although the study was commenced prior to the nomenclature change from non-alcoholic fatty liver disease (NAFLD) to MASLD, all patients recruited also met the criteria for MASLD (steatotic liver disease in the presence of one or more metabolic risk factors). To increase the likelihood of including participants with MASLD and a high intrahepatic triglyceride content, a controlled attenuation parameter (CAP) reading of >305 dB/m confirmed by transient elastography was selected as a threshold for study enrollment. This cut-off has a specificity of 82.5% and a positive predictive value of 80% for the detection of a hepatic magnetic resonance imaging proton density fat fraction (MRI-PDFF) of ≥10%.^15,83^ Age and sex matched control participants were identified from the Oxford Biobank, a database of >9000 volunteers in Oxfordshire who have undergone metabolic phenotyping and consented to be reapproached for clinical research. Controls were screened for the presence of hepatic steatosis using FibroScan and were only included if CAP was <288 dB/m, corresponding with a low likelihood of having a MRI-PDFF ≥5% (sensitivity 75%, specificity 77.1%, PPV 88.7%, and NPV 56.2%).^15^ All study participants (in both groups) were overweight (defined as a BMI ≥25 kg/m^2^) and aged 18–75 years. Exclusion criteria comprised the presence of type 1 or type 2 diabetes, cirrhosis, a diagnosis of obstructive sleep apnea, night-shift working within the last 1-year, pregnancy, combined oral contraceptive pill use, hemoglobin <120 mg/dl, a history of greater than recommended alcohol intake (>14 units/week), or a CAP 288–305 dB/m. Patients with MASLD and a coexisting chronic liver disease of an alternative etiology were also excluded (e.g. chronic viral hepatitis, autoimmune liver disease).

### Method Details

#### Metabolic study visits

All participants underwent identical metabolic profiling during a 6-hour study visit performed during the day (07:00 AM–13:00 PM) and during the night (19:00 PM – 01:00 AM). The order of day and night-time study visits was randomly determined and were separated by an interval of <2-weeks. The sequence of investigations was established at the point of enrollment using a computerized randomization function in R. To maintain unpredictability and prevent repeatable patterns, the randomization process utilized a dynamically generated seed based on the system time. All study visits were performed following a 12-hour fast and the final meal consumed prior to metabolic investigations was calorie matched for each participant (i.e. dinner before daytime study visit and breakfast before night-time study visit). All study procedures were conducted in the same procedure room under ambient electric lighting conditions. For 24-hours prior to commencing each study visit, participants were advised to avoid moderate-strenuous exercise, alcohol intake, and foods with high concentrations of corn-starch which may interfere with stable isotope assessments of glucose utilization. All participants were instructed to maintain habitual eating behaviors prior to day and night-time investigations and the calorie content of the last meal before each visit was matched. Participants were asked to complete a self-reported food diary corresponding to the 7-days prior to day and night-time visits. To minimize the metabolic impact of physical activity immediately prior to attending each study visit, all participants were transported between home and the research unit by taxi and then remained sedentary in bed for 30 minutes prior initiating any study procedures. During the study visit, short periods of napping were permitted but not during the final 30 minutes of the steady-state phases of the clamp. In practice, the majority of participants remained awake throughout the clamp and any meaningful periods of sleep were not achieved due to the need to provide breath samples every 30 minutes.

#### 2-step hyperinsulinemic euglycemic clamp

At 07:00 AM or 19:00 PM for day or night-time visits respectively, arterialized blood was sampled to determine the fasting plasma glucose concentration to be maintained (“clamped”) throughout the entire study period. At the commencement of the two-step hyper-insulinemic euglycemic clamp a bolus of [U-^13^C]-glucose (Cambridge Isotope Laboratories, Andover, USA) was administered (2 mg/kg over 1 min followed by a continuous infusion in 0.9% saline (20 μg/kg/min). Blood glucose was monitored at 15-min intervals during the initial 120 mins (t = 0–120 min, basal phase). At t = 120 min, an insulin infusion (Actrapid; Novo Nordisk) was infused at 10 mU/m^2^/min (low-dose) alongside an infusion of 20% dextrose supplemented with [U-^13^C]-glucose to 4%; blood glucose levels were monitored at 5-min intervals (t = 120–240 min). At t = 240 min, the insulin infusion rate was increased to 50 mU/m2/min (high-dose) and continued for another 120 min (t = 240–360 min) with continued 5-min blood glucose monitoring. Blood samples were taken at 3 time points in the last 30 min of each phase (basal, low- and high-insulin) for steady-state measurements of insulin, endogenous glucose production rate (EGP) and glucose disposal (Rd) calculated using modified versions of the Steele Equations.^64^ M/I values, EGP and Rd were all normalized to DEXA-derived lean mass in order to better reflect glucose handling *in vivo*.^84^ Breath samples were collected at half hourly intervals throughout the 2-step clamp and the generation of ^13^CO_2_ (derived from infused ^13^C-glucose) was measured as a reflection of total body glucose oxidation by calculating the proportion of exhaled tracer relative to the available plasma pool.

#### *De novo* lipogenesis and gluconeogenesis/glycogenolysis

Participants were provided with deuterated water (^2^H_2_O) to drink as two concentrated loading doses 12 hours before attending their study visits. Dilute ^2^H_2_O was then consumed *ad libitum* up until and throughout the 2-step hyperinsulinemic euglycemic clamp. Incorporation of ^2^H from ^2^H_2_O into glucose and very low-density lipoprotein (VLDL) triacylglycerol (TAG)-palmitate was used to determine fractional gluconeogenesis and hepatic *de novo* lipogenesis, respectively.^31^

#### Insulin secretion and clearance

During each stage of the clamp, pre-hepatic insulin secretion rates (ISR) were calculated from deconvolution of plasma C-peptide concentrations using the two-pooled model of Van Cauter et al.^85^ Whole-body insulin clearance during the clamp (CLwb) was calculated as the infusion rate plus endogenous ISR divided by the steady-state plasma insulin concentration.^18,86^ In the basal state, the ratio of basal ISR to basal plasma insulin describes basal CLwb.

#### Adipose and skeletal muscle biopsies

Subcutaneous adipose tissue and skeletal muscle biopsies were sequentially performed in the fasted state prior to commencement of the 2-step hyperinsulinemic euglycemic clamp. Approximately 1g of adipose tissue was sampled by aspirating adipocytes under local anesthetic from the abdominal subcutaneous depot using a Pro-Mag Ultra Biopsy 14g needle. Tissue was immediately washed using 0.9% normal saline to eliminate blood contamination before being placed into liquid nitrogen. A skeletal muscle biopsy was then performed using a spring-loaded gun device inserted vertically through the muscle fascia and into lateral portion of the vastus lateralis. This was repeated up to 3 times to ensure a total of approximately 100 μg of muscle was collected which was then frozen in liquid nitrogen.

#### Plasma proteomics

Plasma proteomic profiling was performed using an Olink Biosciences platform (Uppsala, Sweden). We included proteins from the Explore cardiometabolic I and Inflammation panel which each contained 388 proteins. There were three overlapping proteins contained in both panels and therefore a total of 691 unique proteins were measured. The Olink technology uses an immunoaffinity technique which leverages an extensive collection of nucleotide-labelled antibodies in an immuno-PCR method referred to as a proximity extension assay (PEA). Normalized Protein Expression (NPX), an arbitrary unit on the Log^2^ scale, was used to evaluate protein abundance.

#### UK Biobank replication

The UK Biobank study is a large population-based cohort study of over 500,000 individuals who were recruited at ages 37–73 years from across the UK between 2006 and 2010. The study includes extensive health and lifestyle questionnaire data, physical measures, and biological samples from which genetic data has been generated. The study protocol is available online and more details have been published elsewhere.^20^ At recruitment, the participants gave informed consent to participate and be followed up. UK Biobank has received ethnical approval from the UK National Health Service’s National Research Ethics Service (ref 11/NW/0382). Olink protein measurements were performed as part of the Pharma Proteomic Project (UKB-PPP) on blood plasma samples using the antibody-based protein Olink Explore 3072 Proximity Extension Assay.^87^ Proteomics were generated for 54,219 participants considered to be highly representative of the UK Biobank population on baseline characteristics, enriched for selected diseases (ref). Quality control, sample selection and data processing has been described previously.^87^ Time of blood sample collection was obtained and used to define ‘morning’ (before 10am) and ‘evening’ (after 6pm) samples. International Classification of Disease (ICD)-9 and ICD-10 codes were used to define diagnoses for MASLD (ICD-9 571.8 and ICD-10 K76.0 and K75.8). Exclusions were made for other liver disease diagnoses (alcohol related liver disease, viral hepatitis, autoimmune liver disease, hemochromatosis, Wilson’s disease, alpha-1-antitrypsin deficiency, BuddChiari syndrome) and alcohol use disorder. Associations between time of sampling (morning vs. evening) and Olink protein measures (NPX) were tested via multiple linear regression, adjusted for age, sex, ethnicity, Townsend deprivation index, assessment centre, smoking status, alcohol intake, BMI and duration of fasting.

#### Adipose and skeletal muscle tissue proteomics

Samples were lysed in 6M guanidine containing 100 mM Tris, 10 mM ris(2-carboxyethyl)phosphine and 40 mM 2-chloroacetamide pH 8.5 by tip-probe sonication. The lysate was heated at 95°C and centrifuged at 20,000 x g for 30 min at 4°C. Protein was precipitated with 4 volumes of cold acetone, washed with 80% acetone and resuspended in 10% TFE, 100mM HEPEs pH 7.4. Protein concentration was estimated with BCA, normalized to 10 μg/10 μL and digested with 0.2 μg of trypsin and 0.2μg of LysC overnight at 37°C. One hundred microliters of 99% isopropanol and 1% trifluoracetic acid was added to the digest and peptides purified using inhouse made styrenedivinylbenzene-reverse phase sulfonate microcolumns.

For analysis of adipose tissue, peptides were separated on a Dionex 3500 nanoUHPLC, coupled to an Orbitrap Exploris 480 mass spectrometer via electrospray ionization in positive mode with 1.9 kV at 275 °C and RF set to 30%. Separation is achieved on a 50 cm × 75 μm column packed with C18AQ (1.9 μm) over 75 min at a flow rate of 300 nL/min. Peptides were eluted over a linear gradient of 3%–40% Buffer B (Buffer A: 0.1% v/v formic acid; Buffer B: 80% v/v acetonitrile, 0.1% v/v FA) and the column was maintained at 50°C. The instrument was operated in data-independent acquisition (DIA) mode, with an MS1 spectrum acquired over the mass range 350–1,400 m/z (120,000 resolution, 100% automatic gain control (AGC), and 50 ms maximum injection time) followed by sequential MS/MS spectra across 13.7 m/z isolation windows with 1 m/z overlap covering the full mass range. MS/MS data will be acquired with higher-energy collisional dissociation (HCD) fragmentation (30,000 resolution, 2000% AGC, 55 ms maximum injection time, and normalized collision energy 30%). For analysis of skeletal muscle tissue, peptides were separated on a Vanquish nanoHPLC, coupled to an Orbitrap Astral mass spectrometer via electrospray ionization in positive mode with 1.9 kV at 275 °C and RF set to 50%. Separation is achieved on a 5.5 cm μPAC column over 20 min at a flow rate of 750 nL/min. Peptides were eluted over a linear gradient of 3%–40% Buffer B (Buffer A: 0.1% v/v formic acid; Buffer B: 80% v/v acetonitrile, 0.1% v/v FA). The instrument was operated in data-independent acquisition (DIA) mode, with an MS1 spectrum acquired over the mass range 380–980 m/z (120,000 resolution, 500% automatic gain control (AGC), and 50 ms maximum injection time) followed by sequential MS/MS spectra across 2 m/z isolation windows covering the full mass range. MS/MS data will be acquired with higher-energy collisional dissociation (HCD) fragmentation (500% AGC, 3 ms maximum injection time, and normalized collision energy 27%). Data were processed with Spectronaut v19.0.240604.62635 and searched against the human UniProt Database (March 2023; UP000000589_9606 and UP000000589_9606_additional) using library-free directDIA with default parameters and peptide spectral matches, peptide and protein false discovery rate (FDR) set to 1%. All data were searched with oxidation of methionine and N-terminal protein acetylation set as the variable modification and carbamidomethylation set as the fixed modification. Peptide quantification was carried out at MS2 level using 3–6 fragment ions, with automatic interference fragment ion removal as previously described.^88^ Dynamic mass MS1 and MS2 mass tolerance was enabled, and retention time calibration was accomplished using local (non-linear) regression. A dynamic extracted ion chromatogram window size was performed.

We performed a qualitative comparison of the proteins identified between plasma (Olink) and adipose/muscle (LC-MS/MS). Mapping was based on gene name rather than proteoform analysis. A total of 177 proteins were identified by all 3 methods while 2,935 proteins were identified in both adipose and skeletal muscle but absent in Olink. A total of 415, 1,625 and 819 proteins were uniquely identified in plasma, muscle and adipose respectively.

#### Hepatic transient elastography and body composition assessment

As part of the screening process, all participants received hepatic transient elastography using handheld FibroScan to determine CAP and liver stiffness measurement (LSM). Body weight was recorded at every study visit, and waist circumference and a dual-energy x-ray absorptiometry (DEXA) scan was performed at each day-time study visit to quantify total fat mass, lean mass, and visceral fat mass.

#### Assessment of lifestyle parameters including diet, exercise, and sleep

To account for external factors which may influence metabolism independently of the effect of time-of-day, a range of data on lifestyle parameters were collected corresponding to the period preceding day- and night-time investigations. All participants were asked to keep a self-reported food diary which recorded all energy intake over the 7 days prior to day and night-time study visits. Estimates of calorie content were made from food descriptions using an online calorie calculator^89^ allowing mean daily and meal specific calorie intake to be determined. At each study visit a short-form International Physical Activity Questionnaire (IPAQ) was performed. This generated a metabolic equivalent of task-minutes per week (MET-min/week) which quantifies the intensity and duration of physical activity performed over the preceding 7 days. At the participant’s first study visit a Pittsburgh Sleep Quality Index (PSQI) was used to assesses sleep duration and quality over the preceding one month. At both day and night-time visits, participants were asked to report their sleep onset and wake times over the last week to allow estimation of their sleep mid-point.

#### Biochemical and stable isotope analysis

Fasting biochemical bloods including full blood count, lipid profile, renal function and liver biochemistry were analyzed through NHS clinical laboratories at Oxford University Hospitals NHS Foundation Trust. Serum insulin and C-peptide was measured at baseline and across the 2-step clamp using a commercially available enzyme linked immunosorbent assays (ELISA) (Mercodia, Uppsala, Sweden). Concentrations of glycerol, non-esterified fatty acids (NEFA), TAG, glucose, urea, apolipoprotein B (ApoB), total cholesterol (TC), high-density lipoprotein cholesterol (HDL-C), 3-hydroxybutyrate (3OHB), and lactate were measured using commercially available kits on an ILAB600/ILAB650 clinical analyzer (Instrumentation Laboratory UK, Warrington, UK). Plasma enrichment of ^13^C-glucose was measured using gas chromatography-mass spectrometry to calculate EGP and Rd.

#### Lifestyle and weight loss intervention for patients with MASLD

After baseline day and night-time assessments had been performed, patients with MASLD were enrolled into a 12-week commercially available weight loss and lifestyle program (Slimming World or Weight Watchers). Participants could choose whether to participate in these programs digitally, in person, or a combination of both. A face-to-face or telephone consultation was scheduled after 6–8 weeks to assess compliance with the intervention, address barriers to adherence, and to provide tailored lifestyle advice to help achieve weight loss.

#### Quantification and Statistical Analysis

We powered the study based on anticipated diurnal changes in hepatic de novo lipogenesis (DNL), which served as the primary outcome measure. We have previously measured DNL in healthy participants as 6.3 ± 1.5%.^90^ Given that lipid species exhibit up to 60% diurnal variation across a 24-hour cycle,^91^ we conservatively powered the study to detect a 20% relative change in hepatic DNL between day and night. Assuming an α of 0.05% and 80% power, the required sample size was calculated to be 12 participants in each group. All grouped data is presented as mean ± standard error of mean (SEM) unless otherwise stated. Areas under the curve (AUC) were calculated by the trapezoid method and presented according to the relevant period of the 2-step hyperinsulinemic euglycemic clamp. All data sets were tested for normality according to the Shapiro–Wilk test. Comparisons within the group and between groups were made using a paired or unpaired t-tests or non-parametric equivalents. Data from serial timepoints across the 2-step clamp were compared using a repeated measures ANOVA, with time across the clamp and day/night used as relevant factors to investigate a change. Correlation analyses were carried out using Pearson or Spearman coefficient and non-linear regression analyses were used to plot insulin, 3OHB, and NEFA across the clamp and to calculate concentrations of insulin required to suppress plasma 3OHB or NEFA by 50%. The statistical details of each comparison can be found in the figure legends. Data were analyzed using R, GraphPad Prism 10, and SPSS 25 software. For plasma proteomic analysis, the R package OlinkAnalyze was used to find differentially abundant protein NPX values according to group and time-of-day. Tissue proteomics data were analyzed in Perseus^82^ with log2(x) transformation, and median-based normalization of skeletal muscle data whereas adipose data were first normalized to summed intensity of ribosomal proteins followed by median-based normalization to account for variation in the amount of blood contamination in human adipose biopsies as previously described.^92^ Differential abundance was calculated with paired Student’s t-test (day verses night) and q-values generated using Benjamini-Hochberg with FDR set to 5%.

## Supplementary Material

STROBE checklist

Supplemental information

Supplementary Data S1

Supplementary Table S9

Supplementary Table S10

Supplementary Table S11

Supplementary Table S12

Supplementary Table S13

Supplementary Table S14

Supplementary Table S15

## In brief

By studying metabolism across day and night in human participants, Marjot et al. show that MASLD is a nighttime disease driven by poor insulin action and low insulin levels. These daily patterns persist after weight loss, suggesting that nighttime metabolic dysfunction is a key driver of liver fat accumulation.

## Highlights

- MASLD is driven by hepatic and peripheral insulin resistance, specifically at night
- In MASLD, there is low nighttime insulin secretion and high insulin clearance
- Nighttime metabolic dysfunction persists despite weight loss and liver fat reductions
- This will inform the optimal window for energy intake, exercise, and medication delivery

## Resource Availability

### Lead contact

Requests for further information and resources should be directed to and will be fulfilled by the lead contact, Jeremy W. Tomlinson (jeremy.tomlinson@ocdem.ox.ac.uk).

### Materials availability

The study did not generate new, unique reagents.

### Data and code availability

The tissue proteomics data have been deposited to the ProteomeXchange Consortium via the PRIDE partner repository with the dataset identifier PXD061275. Underlying data to generate the graphs in the manuscript can be found in Data S1. This study did not generate any code. Any additional information required to reanalyze the data reported in this paper is available from the lead contact upon request
