# Supplementary material for: Human MASLD is a diurnal disease driven by multisystem insulin resistance and reduced insulin availability at night
Source: Cell Metab. Author manuscript; Available in PMC 2026 Aug 26. (PMC7619395; doi:10.1016/j.cmet.2025.12.004)
Supplement: Supplementary file 2 [file EMS217802-supplement-Supplemental_information.pdf]

Cell Metabolism, Volume *38*

Supplemental information

Human MASLD is a diurnal disease driven by multisystem insulin resistance and reduced insulin availability at night

Thomas Marjot, Kieran Smith, Felix Westcott, Sarah White, Elspeth Johnson, Nikola Srnic, Amy Barrett, Ellis Hall, Kate Gralton, Kaitlyn Dennis, Hamish Miller, Riccardo Pofi, Jeremy F.L. Cobbold, Rebecca Richmond, Fredrik Karpe, Ronnie Blazev, Matthew J. Watt, Benjamin L. Parker, Leanne Hodson, David W. Ray, and Jeremy W. Tomlinson

Table S1

	MASLD BL (n=12)	Ctrl (n=12)	MASLD FU (n=11)	P value† MASLD BL vs. Ctrl	P value* MASLD BL vs. FU	P value† MASLD FU vs. Ctrl
**Demographics**						
Age (years)	49.0 (4.2)	58.6 (2.6)	51.6 (3.6)	0.0679	N/A	0.1339
Male sex, n (%)	8 (67%)	5 (42%)	8 (73%)	0.4126	N/A	0.2801
**Metabolic parameters**						
Weight (kg)	106.4 (4.4)	82.4 (2.9)	99.0 (4.6)	**0.0002**	**0.0030**	**0.0074**
BMI (kg/m^2)	35.5 (1.6)	27.5 (0.5)	33.0 (1.8)	**0.0004**	**0.0037**	**0.0138**
Body fat (%)	42.0 (1.5)	33.7 (2.6)	38.4 (1.9)	**0.0116**	**0.0006**	0.1550
Total fat mass (kg)	43.1 (2.8)	26.1 (1.7)	40.6 (3.7)	**0.0001**	0.6176	**0.0030**
Lean mass (kg)	58.7 (2.2)	52.5 (3.4)	52.8 (5.6)	0.1404	0.3592	0.9638
Visceral fat (kg)	2.8 (0.4)	0.8 (0.2)	2.4 (0.3)	**0.0001**	**0.0314**	**0.0001**
Waist circumference (cm)	117.3 (3.6)	95.7 (1.6)	110.7 (4.1)	**0.0001**	**0.0000**	**0.0046**
PSQI	7.2 (1.4)	4.8 (0.8)	6.2 (1.6)	0.1612	0.1332	0.4344
**Laboratory tests**						
Haemoglobin (g/L)	146.4 (3.4)	138.0 (2.6)	135.8 (3.7)	0.0662	**0.0281**	0.6367
Platelet count (×10^9/L)	258.6 (19.2)	247.0 (14.4)	249.4 (21.9)	0.6347	**0.0666**	0.9283
Bilirubin (µmol/L)	12.8 (2.2)	9.7 (1.5)	10.0 (1.5)	0.2507	0.1461	0.8759
ALT (U/L)	47.4 (9.1)	23.3 (2.9)	22.5 (3.0)	**0.0253**	**0.0106**	0.8595
AST (U/L)	32.0 (6.4)	31.0 (3.4)	19.7 (1.6)	0.8910	**0.0409**	**0.0088**
γGT (U/L)	35.6 (2.3)	25.8 (3.6)	24.4 (2.2)	**0.0339**	**0.0012**	0.7393
ALP (U/L)	73.3 (5.3)	68.3 (3.4)	72.4 (6.1)	0.4300	0.4353	0.5637
Serum albumin (g/L)	38.2 (0.7)	40.8 (0.8)	37.0 (0.7)	**0.0219**	0.1544	**0.0022**
Creatinine (µmol/L)	68.7 (7.7)	81.4 (3.9)	76.0 (8.2)	0.1586	**0.0380**	0.5619
eGFR (mL/min/1.73m^2)	85.8 (4.2)	79.9 (3.7)	84.0 (4.8)	0.3011	0.4402	0.5104
INR	1.0 (0.0)	1.0 (0.0)	1.0 (0.0)	1.0000	0.5911	0.7357
HbA1c (%)	5.8 (0.2)	5.4 (0.1)	5.8 (0.1)	**0.0497**	0.2889	**0.0117**
HbA1c (mmol/mol)	40.2 (2.1)	35.5 (0.9)	40.1 (1.4)	0.0559	0.3213	**0.0148**
IGT, n (%)	6 (50%)	0 (0%)	5 (46%)	**0.0184**	1.0000	**0.0328**
Total cholesterol (mmol/L)	4.6 (0.4)	5.7 (0.3)	4.7 (0.2)	**0.0285**	0.2156	**0.0114**
HDL-C (mmol/L)	1.0 (0.1)	1.7 (0.1)	1.1 (0.1)	**0.0004**	1.0000	**0.0010**
LDL-C (mmol/L)	3.0 (0.3)	3.6 (0.3)	2.9 (0.2)	0.1506	**0.0182**	0.0915
Triglycerides (mmol/L)	1.5 (0.2)	1.3 (0.3)	1.4 (0.2)	0.4368	0.3216	0.6899
**Transient elastography**						
CAP (dB/m)	338.8 (7.0)	238.8 (7.3)	278.7 (16.9)	**0.0000**	**0.0078**	**0.0478**
LSM (kPa)	7.0 (0.6)	4.6 (0.6)	7.1 (0.7)	**0.0070**	0.7789	**0.0092**

Supplementary table 1. Clinical characteristics of MASLD group at baseline (BL), control participants (Ctrl), and MASLD group at follow up (FU), relating to Fig 1.

Data are represented as mean ± standard error unless otherwise stated. Aggregate data is presented for all available participants in each group (n=12 in MASLD BL, n=12 in Ctrl, n=11 in MASLD FU). However, paired comparisons were conducted only for participants with both BL and FU measurements (n=11) to account for one MASLD patient who dropped-out after baseline investigations. *Paired t-tests (between patients with MASLD at BL and FU. †Unpaired t-tests between patients with MASLD (BL/FU) and Ctrl. Chi-squared test was used to determine differences in proportions of male sex and IGT between groups. Boldfaced p-values are significant (<0.05). BMI: body mass index, PSQI: Pittsburgh Sleep Quality Index; ALT: alanine transaminase, AST: aspartate transferase, γGT: gamma-glutamyl transferase, ALP: alkaline phosphatase, INR: international normalised ratio, HbA1c: glycated haemoglobin, IGT: impaired glucose tolerance (HbA1c 42-48 mmol/mol), HLD-C: high density lipoprotein cholesterol, LDL-C: low density lipoprotein cholesterol, CAP: controlled attenuation parameter, LSM: liver stiffness measurement.

Table S2

	Total BL MASLD cohort (n=12)			Sensitivity analysis removing x1 patient (21 yrs)		
**Metabolic parameter**	**Day Mean (SE)**	**Night Mean (SE)**	**P value**	**Day Mean (SE)**	**Night Mean (SE)**	**P value**
**M/I value [low insulin]**	6.67 (3.49)	3.45 (1.15)	**0.0210**	6.84 (1.09)	1.36 (1.26)	**0.0244**
**EGP [low insulin]**	12.7 (0.83)	14.3 (0.99)	**0.0251**	12.9 (0.86)	14.7 (1.00)	**0.0299**
**Rd [high insulin]**	46.1 (3.5)	34.0 (2.98)	**0.0008**	47.3 (3.60)	34.9 (3.11)	**0.0015**
**AUC NEFA [basal]**	20,708 (934)	24,970 (815)	**0.0001**	20,825	24,688	**0.0002**
**AUC NEFA [low insulin]**	7659 (626)	10,851 (994)	**0.0002**	7570 (679)	10,634 (1062)	**0.0007**
**AUC 3OHB [basal]**	3339 (777)	9180 (1760)	**0.0010**	3405 (848)	9338 (1920)	**0.0020**

Supplementary table 2. Sensitivity analysis of MASLD BL group before and after removing a single young patient (21 years), relating to Fig. 1-4.

Data are represented as mean ± standard error unless otherwise stated. Aggregate data is presented for all available participants in each group. Units: M/I values: mg/kg lean mass/min per mU/L insulin *100, EGP: µmol/kg lean mass/min, Rd: µmol/kg lean mass/min, AUC NEFA: µmol/L, AUC OHB: µmol/L

Table S3

	Total Ctrl cohort (n=12)			Sensitivity analysis removing x1 menstruating patient		
**Metabolic parameter**	**Day Mean (SE)**	**Night Mean (SE)**	**P value**	**Day Mean (SE)**	**Night Mean (SE)**	**P value**
**M/I value [low insulin]**	20.1 (3.50)	13.5 (2.74)	0.1508	20.4 (3.83)	12.8 (2.88)	0.1268
**EGP [low insulin]**	13.6 (1.05)	12.6 (0.88)	0.2334	13.8 (1.12)	12.9 (0.91)	0.3652
**Rd [high insulin]**	95.9 (7.61)	76.6 (6.57)	**0.0062**	95.6 (8.33)	73.6 (6.40)	**0.0027**
**AUC NEFA [basal]**	16,830 (1970)	25,345 (2440)	**0.0016**	17,324 (2090)	25,881 (2607)	**0.0034**
**AUC NEFA [low insulin]**	2770 (558)	3708 (642)	0.2661	2947 (580)	3952 (651)	0.2783
**AUC 3OHB [basal]**	2284 (424)	10,779 (2333)	**0.0015**	2378 (453)	10,111 (2449)	**0.0029**

Supplementary table 3. Sensitivity analysis of Ctrl group before and after removing a single premenopausal participant with active menstrual cycles, relating to Fig. 1-4.

Data are represented as mean ± standard error unless otherwise stated. Aggregate data is presented for all available participants in each group. Units: M/I values: mg/kg lean mass/min per mU/L insulin *100, EGP: µmol/kg lean mass/min, Rd: µmol/kg lean mass/min, AUC NEFA: µmol/L, AUC OHB: µmol/L

Table S4

	MASLD BL day		MASLD BL night				Ctrl BL day		Ctrl BL night			
	**Mean (SD)**	**Variance**	**Mean (SD)**	**Variance**	**F statistic**	**P value**	**Mean (SD)**	**Variance**	**Mean (SD)**	**Variance**	**F statistic**	**P value**
M/I value [low ins]	6.7 (3.5)	12.2	3.5 (4.0)	15.9	1.30	0.3322	20.1 (12.1)	147.3	13.5 (9.5)	90.2	1.63	0.2146
M/I value [high ins]	5.7 (4.1)	5.9	4.1 (2.2)	4.8	1.20	0.3689	15.3 (5.3)	28.4	12.8 (4.0)	15.9	1.78	0.1764
EGP [basal]	21.9 (6.32)	40.0	19.5 (3.2)	10.5	3.80	**0.0182**	23.5 (3.4)	11.5	19.8 (3.5)	12.4	1.08	0.4515
EGP [low insulin]	12.7 (2.9)	8.36	14.3 (3.4)	11.7	1.40	0.2913	13.6 (3.6)	13.2	12.6 (3.0)	9.3	1.42	0.2839
EGP [high ins]	7.1 (4.0)	15.9	8.2 (3.4)	11.8	1.35	0.3139	9.8 (5.3)	28.6	8.8 (3.9)	15.2	1.88	0.1557
Rd [basal]	22.8 (6.7)	44.2	19.7 (3.1)	9.6	4.61	**0.0089**	23.8 (3.6)	13.0	20.3 (3.7)	14.0	1.07	0.4539
Rd [low ins]	24.8 (3.8)	14.4	19.1 (2.0)	4.0	3.65	**0.0211**	35.7 (13.0)	168.9	26.0 (8.4)	70.7	2.39	0.0820
Rd [high ins]	46.1 (12.2)	148.8	34.0 (10.3)	106.6	1.40	0.2950	95.9 (26.4)	694.8	76.6 (22.8)	518.4	1.34	0.3178
Rd/I [high ins]	0.37 (0.14)	0.02	0.31 (0.12)	0.01	1.51	0.2532	0.97 (0.30)	0.09	0.74 (0.23)	0.05	1.70	0.1967
AUC NEFA [basal]	20708 (3235)	$1.1*10^{7}$	24970 (2823)	$8.0*10^{6}$	1.30	0.3295	16830 (6826)	$4.7*10^{7}$	25345 (8451)	$7.1*10^{7}$	1.53	0.2451
AUC NEFA [low ins]	7659 (2168)	$4.7*10^{6}$	10851 (3443)	$1.2*10^{7}$	2.52	0.0701	2770 (1933)	$3.7*10^{6}$	3708 (2225)	$5.0*10^{6}$	1.32	0.3245
AUC NEFA [high ins]	3223 (1255)	$1.6*10^{6}$	4213 (2213)	$4.9*10^{6}$	3.11	**0.0363**	1697 (1025)	$1.1*10^{6}$	2477 (1622)	$2.6*10^{6}$	2.50	0.0716
AUC OHB [basal]	3339 (2691)	$7.3*10^{6}$	9180 (6096)	$3.7*10^{7}$	5.13	**0.0058**	2284 (1469)	$2.2*10^{6}$	10779 (8082)	$6.5*10^{7}$	30.3	**<0.0001**
AUC OHB [low ins]	925 (407)	$1.7*10^{5}$	1553 (882)	$7.8*10^{5}$	4.69	**0.0082**	489 (196)	$3.9*10^{4}$	743 (518)	$2.7*10^{5}$	6.95	**0.0016**
AUC OHB [high ins]	609 (364)	$1.3*10^{5}$	665 (396)	$1.6*10^{5}$	1.19	0.3913	395 (243)	$5.9*10^{4}$	434 (166)	$2.7*10^{4}$	2.17	0.1077
DNL	11.0 (7.8)	60.1	11.6 (6.7)	44.9	1.34	0.33591	8.8 (7.3)	52.5	4.0 (3.2)	10.3	5.1	**0.0059**

Supplementary table 4. Within group variance across key metabolic parameters, relating to Fig. 1-4.

Data are represented as mean ± standard error unless otherwise stated. Aggregate data is presented for all available participants in each group (n=12 in MASLD BL, n=12 in Ctrl). Units: M/I values: mg/kg lean mass/min per mU/L insulin *100, EGP: µmol/kg lean mass/min, Rd: µmol/kg lean mass/min, Rd/I: µmol/kg lean mass/min per mU insulin, AUC NEFA: µmol/L, AUC OHB: µmol/L, DNL: percentage.

Table S5

	Fasting plasma glucose	Plasma glucose [basal phase] (mmol/L)		Plasma glucose [low insulin] (mmol/L)		Plasma glucose [high insulin] (mmol/L)	
	Mean (SD)	Mean (SD)	CoV (%)	Mean (SD)	CoV (%)	Mean (SD)	CoV (%)
**Ctrl day**	4.65 (0.49)	4.65 (0.53)	3.3	4.55 (0.49)	5.8	4.65 (0.50)	10
**Ctrl night**	4.37 (0.37)	4.36 (0.51)	3.0	4.3 (0.37)	3.9	4.27 (0.38)	10
**MASLD day**	5.57 (0.92)	4.94 (0.64)	3.1	5.53 (0.94)	3.6	5.53 (0.89)	6.5
**MASLD night**	4.22 (0.28)	4.16 (0.44)	1.8	4.31 (0.26)	3.1	4.07 (0.24)	4.1

Supplementary table 5. Plasma glucose concentrations across the 2-step hyperinsulinemia euglycaemic clamp, relating to Fig. 1.

Data are presented as mean ± standard deviation. Co-efficient of variation is representative of replicates taken over the last 30 minutes ('steady state') of each clamp phase (basal x3 samples [every 15 minutes]; low and high insulin x7 samples [every 5 minutes]. Aggregate data is presented for all available participants in each group (n=12 MASLD BL, n=12 Ctrl)

Table S6

	GIR (ml/hr) [low insulin]		GIR (ml/hr) [high insulin]	
	Mean (SD)	CoV	Mean (SD)	CoV
**Ctrl day**	62.1 (9.2)	14.9%	223.3 (13.8)	6.2%
**Ctrl night**	36.4 (1.1)	3.0%	190.7 (11.0)	10.0%
**MASLD day**	35.7 (3.7)	10.4%	123.7 (5.6)	4.7%
**MASLD night**	14.9 (1.0)	6.6%	80.1 (6.6)	8.2%

Supplementary table 6. Glucose infusion rate (GIR) required to maintain euglycaemia across the 2-step hyperinsulinaemic euglycaemic clamp, relating to Fig. 1.

Data are presented as mean ± standard deviation (SD). Co-efficient of variation (CoV) is representative of 3 replicates taken over the last 30 minutes ('steady state') of each clamp phase. Aggregate data is presented for all available participants in each group (n=12 MASLD BL, n=12 Ctrl). Mean GIR data calculated from data presented in **Figure 1C**.

Table S7

	MASLD					
	Day			Night		
**Participant**	**EGP**	**GNG**	**GLY**	**EGP**	**GNG**	**GLY**
**1**	21.69	4.30	17.39	19.29	7.57	11.72
**2**	38.07	13.65	24.43	24.37	10.32	14.06
**3**	22.13	7.44	14.69	16.97	9.23	7.74
**4**	23.23	7.64	15.59	19.11	8.30	10.82
**5**	14.26	5.61	8.66	17.41	6.81	10.60
**6**	17.91	10.42	7.48	18.04	9.77	8.27
**7**	22.26	9.86	12.40	22.37	7.37	15.00
**8**	13.81	5.07	8.74	13.72	5.55	8.17
**9**	24.70	7.69	17.01	20.45	10.48	9.96
**10**	21.09	6.62	14.47	24.26	10.86	13.41
**11**	25.35	7.42	17.93	21.22	11.16	10.07
**12**	17.91	5.82	12.09	16.24	8.70	7.55
**Mean (SE)**	21.87 (1.83)	7.63 (0.75)	14.24 (1.38)	19.45 (0.93)	8.84 (0.51)	10.61 (0.73)

	Ctrl					
	Day			Night		
**Participant**	**EGP**	**GNG**	**GLY**	**EGP**	**GNG**	**GLY**
**1**	17.23	4.96	12.27	17.55	5.77	11.79
**2**	24.34	8.47	15.87	21.38	6.58	14.80
**3**	19.38	8.10	11.27	18.51	6.39	12.11
**4**	20.86	6.36	14.49	20.85	7.48	13.38
**5**	24.13	6.33	17.81	19.19	5.54	13.64
**6**	29.13	1.69	27.44	17.80	6.85	10.95
**7**	25.53	7.87	17.66	16.97	3.17	13.81
**8**	21.40	5.49	15.91	13.99	3.00	10.99
**9**	24.87	6.44	18.43	27.03	13.69	13.34
**10**	26.86	3.31	23.55	21.82	6.77	15.05
**11**	21.97	7.49	14.48	18.31	7.31	11.00
**12**	26.06	7.52	18.54	24.44	9.54	14.90
**Mean (SE)**	23.48 (0.98)	6.17 (0.59)	17.31 (1.31)	19.82 (1.02)	6.84 (0.81)	12.98 (0.45)

Supplementary table 7. Individual values for basal endogenous glucose production (EGP), gluconeogenesis (GNG), and glycogenolysis (GLY), relating to Fig 1.

All data is presented as μmol/kg lean mass/min.

Table S8

	Plasma insulin concentration across 2-step clamp (pmol/L)			
	Fasting	Basal phase	Low dose insulin	High dose insulin
**Ctrl day**	19.4 (2.0)	20.1 (1.8)	115.8 (5.1)	598.7 (24.4)
**Ctrl night**	10.1 (1.9)	15.8 (1.7)	111.4 (6.1)	626.7 (24.2)
**MASLD day**	121.5 (38.5)	127.2 (39.6)	309.1 (73.8)	817.1 (75.9)
**MASLD night**	103.0 (39.0)	86.0 (22.0)	186.0 (34.6)	677.5 (42.2)

Supplementary table 8. Plasma insulin concentrations across the 2-step hyperinsulinaemic euglycaemic clamp, relating to Fig. 5.

Data are presented as mean ± standard error. Aggregate data is presented for all available participants in each group (n=12 in MASLD BL, n=12 Ctrl).

Figure S1.

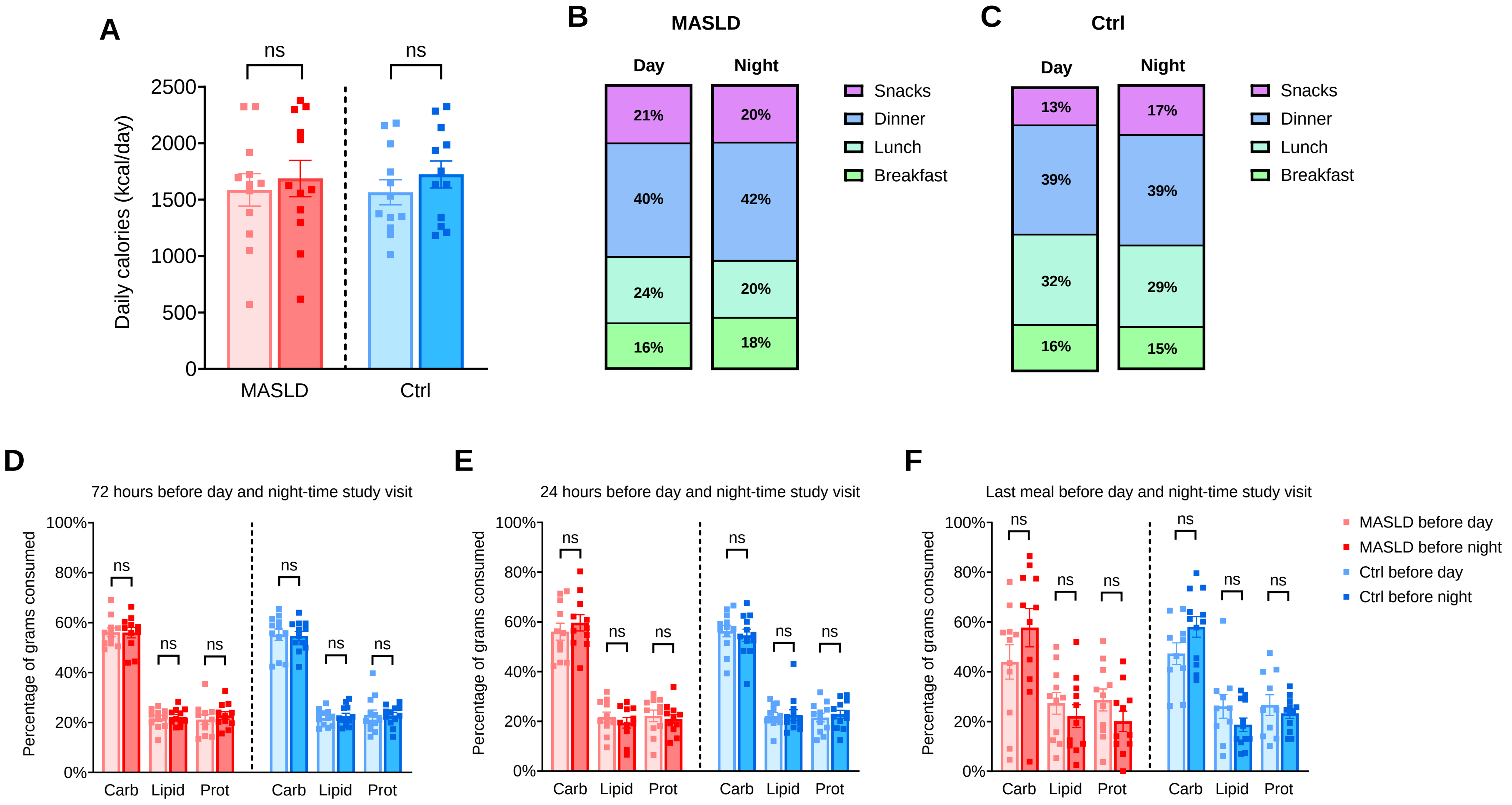

Supplementary figure 1. Dietary composition and distribution of daily calorie intake before day and night-time study visits, relating to Fig. 1 and STAR methods (study protocol).

(A) Mean daily self-reported calorie intake in week preceding day- and night-time study visits

(B) Distribution of daily calorie intake in MASLD group during the 7-days before day and night-time study visits separated by breakfast, lunch, dinner, and snacks

(C) Distribution of daily calorie intake in Ctrl group during the 7-days before day and night-time study visits separated by breakfast, lunch, dinner, and snacks

(D) Dietary composition as percentage of carbohydrates, lipid, and protein consumed 72 hours before day and night-time study visits

(E) Dietary composition as percentage of carbohydrates, lipid, and protein consumed 24 hours before day and night-time study visits

(F) Dietary composition as percentage of carbohydrates, lipid, and protein consumed in the last meal before day and night-time study visits

Data are represented as mean ± SEM. Data represent n = 11 in MASLD and 12 in Ctrl. Non-significant p values were determined by paired t-tests (A-C).

Figure S2.

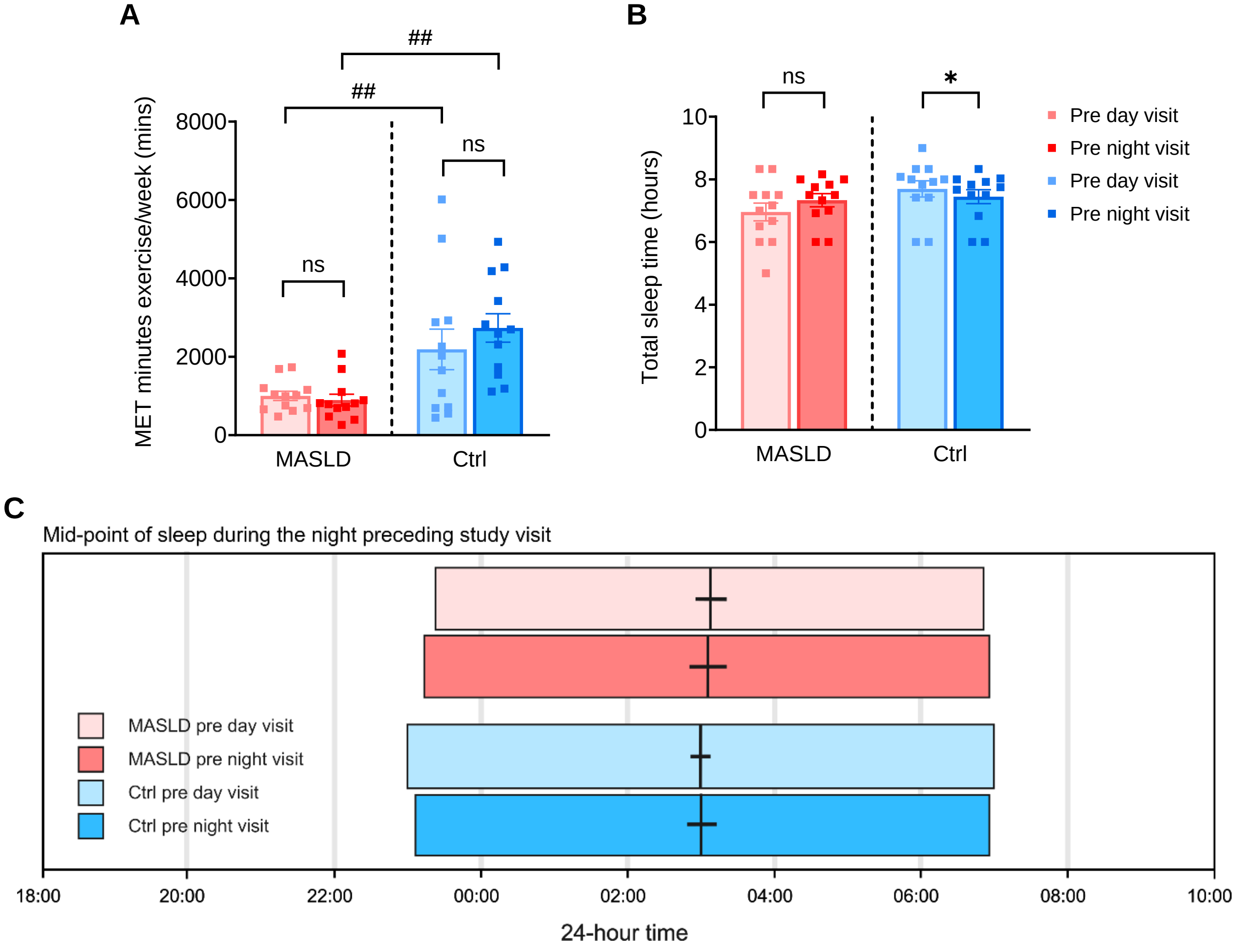

Supplementary figure 2. Data relating to physical activity, calorie intake, and sleep preceding day- and night-time study visits, relating to Fig. 2 and STAR Methods (study protocol).

(A) Metabolic equivalent of task (MET) minutes of exercise in week preceding day- and night-time study visits
(B) Mean total sleep time in week preceding day- and night-time study visits
(C) Mean self-reported sleep onset and mean wake time represented by coloured bars, with mean mid-point of sleep on night prior to day- and night-time study visits

Data are represented as mean ± SEM. Data represent n = 12 in MASLD and 12 in Ctrl. P values were determined by paired (*) or unpaired (#) t-tests with Welch correction.

Figure S3.

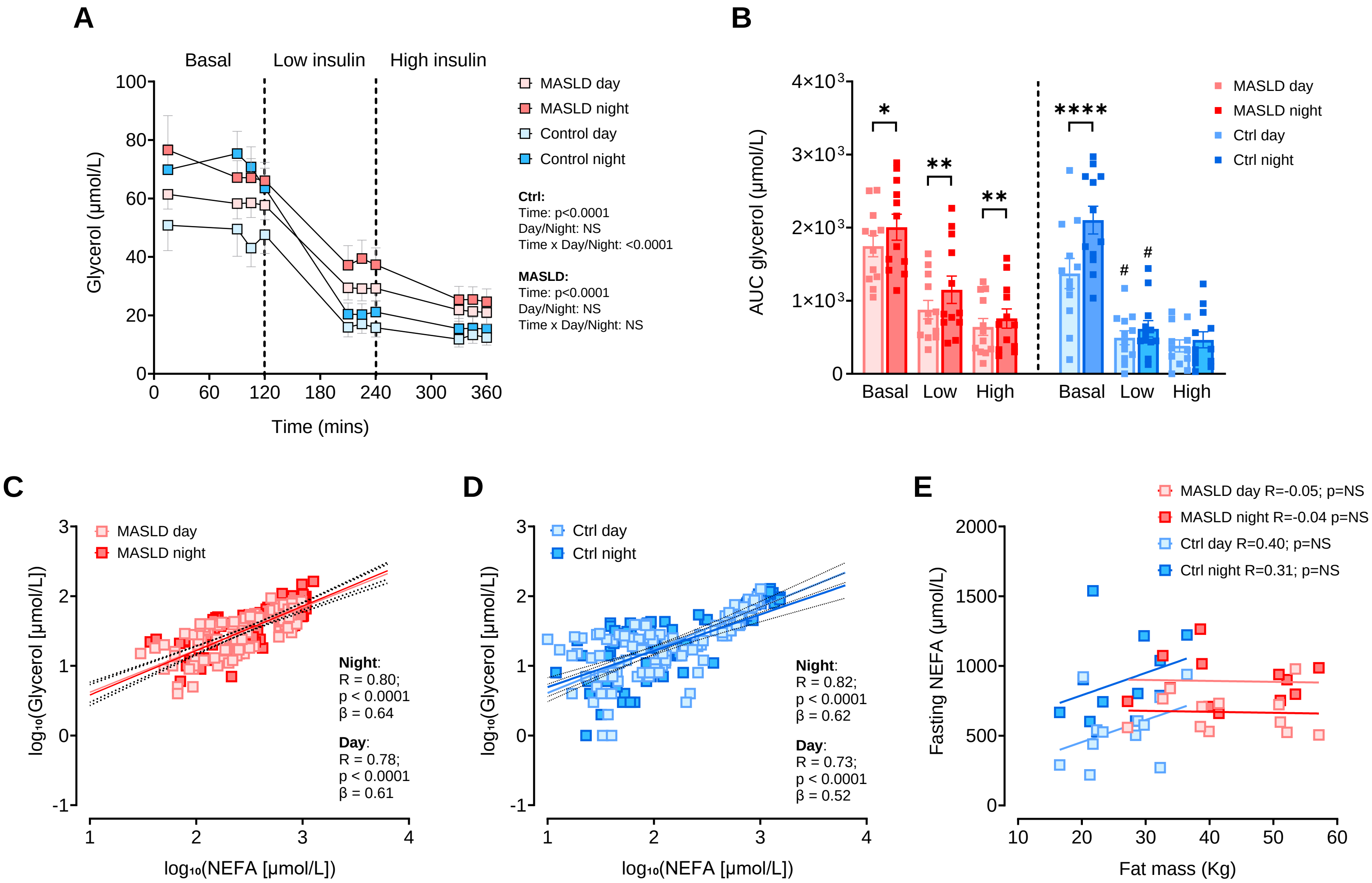

Supplementary figure 3. Dynamics of plasma glycerol across the 2-step clamp and correlations with NEFA, relating to Fig. 3

(A) Trajectory of plasma glycerol across the 2-step clamp during day and night in patients with MASLD and controls (Ctrl).
(B) Area under the curve (AUC) for glycerol during basal, low, and high dose insulin phases of the 2-step clamp.
(C) Correlation between plasma NEFA and Glycerol in MASLD group according to day/night
(D) Correlation between plasma NEFA and Glycerol in Ctrl group according to day/night
(E) Correlations between fat mass and fasting plasma NEFA concentrations

Data are represented as mean ± SEM. n = 12 in MASLD and 12 in Ctrl group. P values were determined by two-way ANOVA tests (A) or paired (*) or unpaired (#) t-tests with Welch correction (B). Correlation analysis was performed using Spearman correlation co-efficient and linear regression (C-E).

Figure S4.

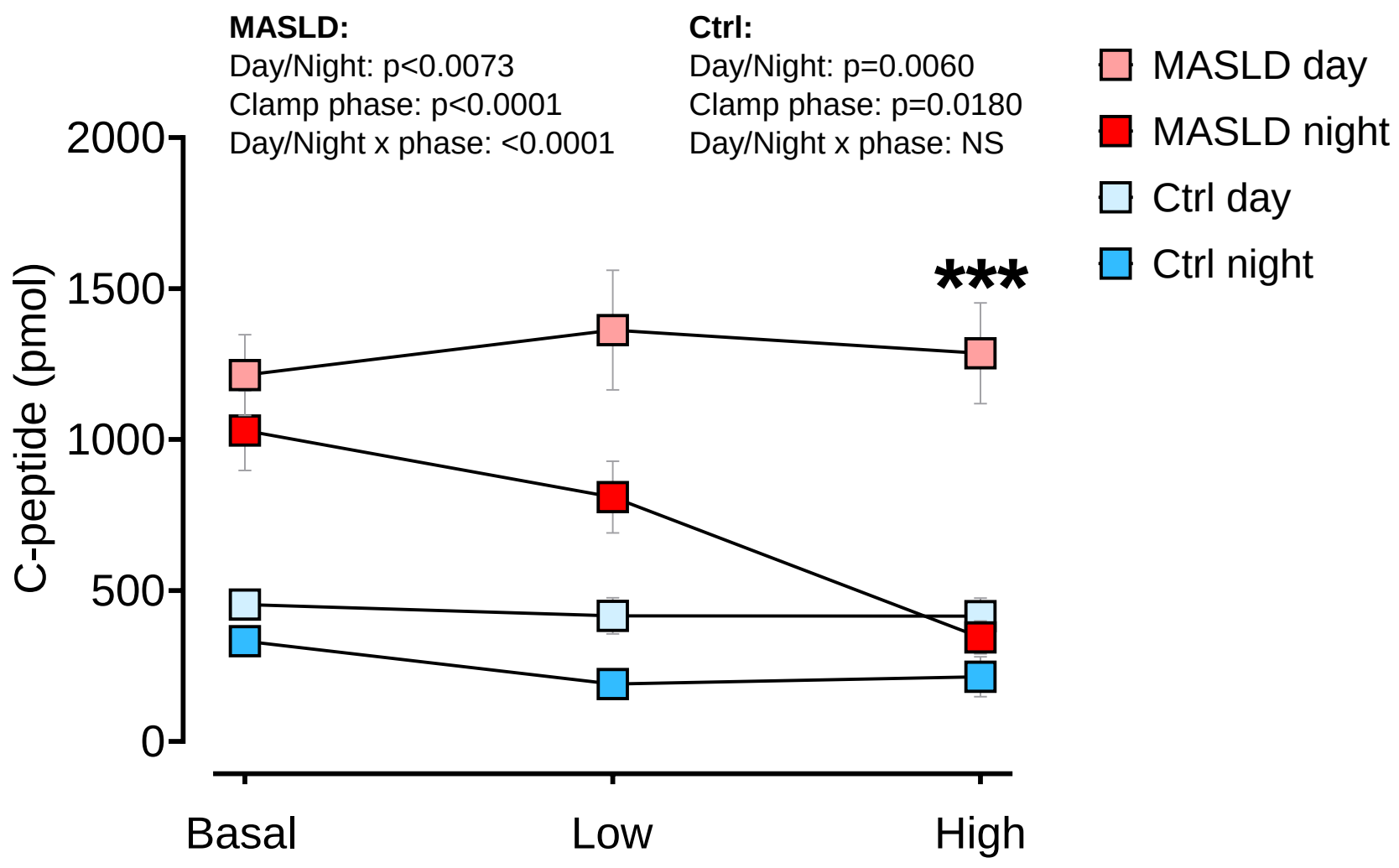

Supplementary figure 4. Concentrations of plasma C-peptide across 2-step hyperinsulinaemic euglycaemic clamp, relating to Fig. 5

Data are represented as mean ± SEM. n = 12 in MASLD and 12 in Ctrl group. P values were determined by two-way ANOVA tests with Šidák multiple comparisons.

Figure S5.

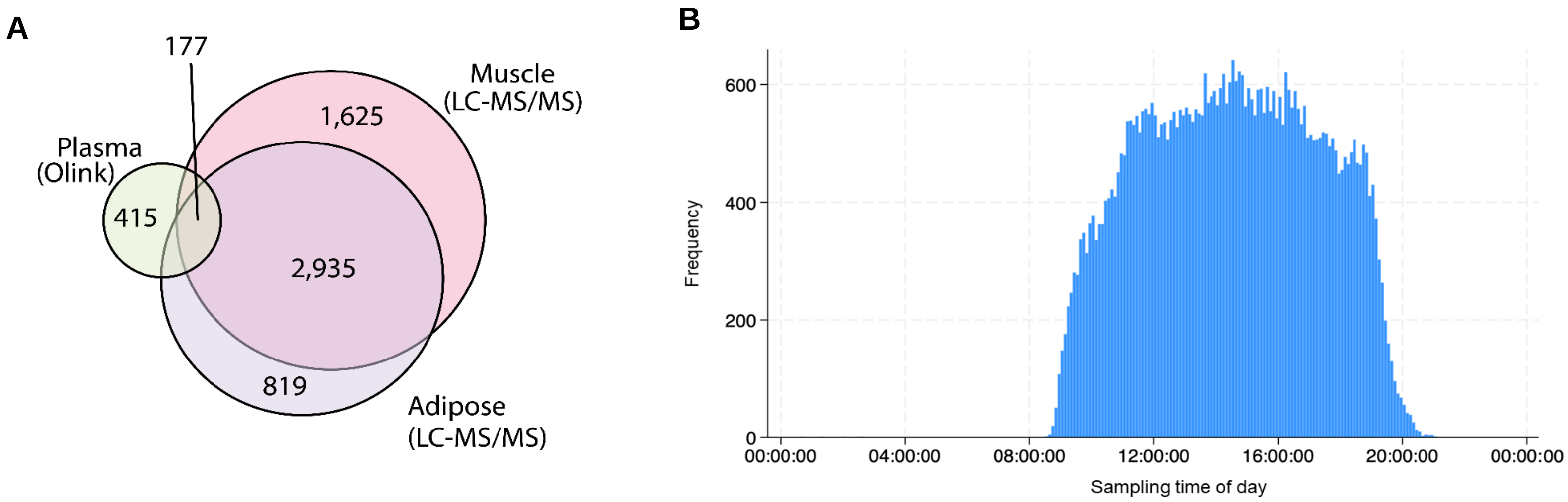

Supplementary figure 5. Additional proteomics methods and results, relating to Fig. 6

(A) Total number and overlapping number of proteins identified in plasma, skeletal muscle, and adipose tissue
(B) Histogram showing time of blood sampling for plasma proteomics (Olink) in UK biobank
